# Influence of Human Activities on Broad-Scale Estuarine-Marine Habitats Using Omics-Based Approaches Applied to Marine Sediments

**DOI:** 10.3390/microorganisms7100419

**Published:** 2019-10-04

**Authors:** Rohan M. Shah, Joseph Crosswell, Suzanne S. Metcalfe, Geoffrey Carlin, Paul D. Morrison, Avinash V. Karpe, Enzo A. Palombo, Andy D.L. Steven, David J. Beale

**Affiliations:** 1 Department of Chemistry and Biotechnology, Faculty of Science, Engineering and Technology, Swinburne University of Technology, P. O. Box 218, Hawthorn, VIC 3122, Australia; rshah@swin.edu.au (R.M.S.); epalombo@swin.edu.au (E.A.P.); 2 Oceans and Atmosphere, Commonwealth Scientific and Industrial Research Organization, P. O. Box 2583, Dutton Park, QLD 4001, Australia; joey.crosswell@csiro.au (J.C.); geoffrey.carlin@csiro.au (G.C.);; 3 Land and Water, Commonwealth Scientific and Industrial Research Organization, P. O. Box 2583, Dutton Park, QLD 4001, Australia; suzanne.metcalfe@csiro.au (S.S.M.); avinash.karpe@csiro.au (A.V.K.); 4 Australian Centre for Research on Separation Science, School of Applied Sciences, RMIT University, Melbourne, VIC 3001, Australia; paul.morrison@rmit.edu.au

**Keywords:** community metabolomics, 16S rRNA gene sequencing, chemometrics, marine ecosystems, sediments, natural environment

## Abstract

Rapid urban expansion and increased human activities have led to the progressive deterioration of many marine ecosystems. The diverse microbial communities that inhabit these ecosystems are believed to influence large-scale geochemical processes and, as such, analyzing their composition and functional metabolism can be a means to assessing an ecosystem’s resilience to physical and chemical perturbations, or at the very least provide baseline information and insight into future research needs. Here we show the utilization of organic and inorganic contaminant screening coupled with metabolomics and bacterial 16S rRNA gene sequencing to assess the microbial community structure of marine sediments and their functional metabolic output. The sediments collected from Moreton Bay (Queensland, Australia) contained low levels of organic and inorganic contaminants, typically below guideline levels. The sequencing dataset suggest that sulfur and nitrite reduction, dehalogenation, ammonia oxidation, and xylan degradation were the major metabolic functions. The community metabolites suggest a level of functional homogeneity down the 40-cm core depth sampled, with sediment habitat identified as a significant driver for metabolic differences. The communities present in river and sandy channel samples were found to be the most active, with the river habitats likely to be dominated by photoheterotrophs that utilized carbohydrates, fatty acids and alcohols as well as reduce nitrates to release atmospheric nitrogen and oxidize sulfur. Bioturbated mud habitats showed overlapping faunal activity between riverine and sandy ecosystems. Nitrogen-fixing bacteria and lignin-degrading bacteria were most abundant in the sandy channel and bioturbated mud, respectively. The use of omics-based approaches provide greater insight into the functional metabolism of these impacted habitats, extending beyond discrete monitoring to encompassing whole community profiling that represents true phenotypical outputs. Ongoing omics-based monitoring that focuses on more targeted pathway analyses is recommended in order to quantify the flux changes within these systems and establish variations from these baseline measurements.

## 1. Introduction

Despite Australia’s vast landmass, an estimated 85% of the total population live within the coastal zone [1]. Ongoing rapid urban expansion, strong industrialization, and modern agriculture have caused intense degradation of coastal riparian vegetation [2], which in turn has led to the progressive deterioration of natural ecosystems [3], and riverine and estuarine water quality [4]. Furthermore, increased human activities have significantly changed the nutrient flux into these aquatic ecosystems [5]. Excessive levels of inorganic nutrients such as nitrogen and phosphorus accelerate the eutrophication of waterways and estuaries [6]. Eutrophication is one of the biggest threats to coasts and estuaries globally [5], causing severe degradation of water quality, loss of biodiversity and richness, ecological shifts, and ecosystem malfunction [7]. Such changes are also likely to adversely affect coastal communities in terms of employment and tourism opportunities and recreational activities. Compounding the issue further, in areas of extreme rainfall, unpredictable flood events drastically alter the quality of run-off and, therefore, delivery of catchment-derived sediments and nutrients [8]. A substantial number of other pollutants such as trace metals, pesticides, and hydrocarbons have also been introduced into coastal waters as a result of human activities, increased urbanization, and as a consequence of greater impervious surfaces constructed within catchments [9].

**Figure 1:**
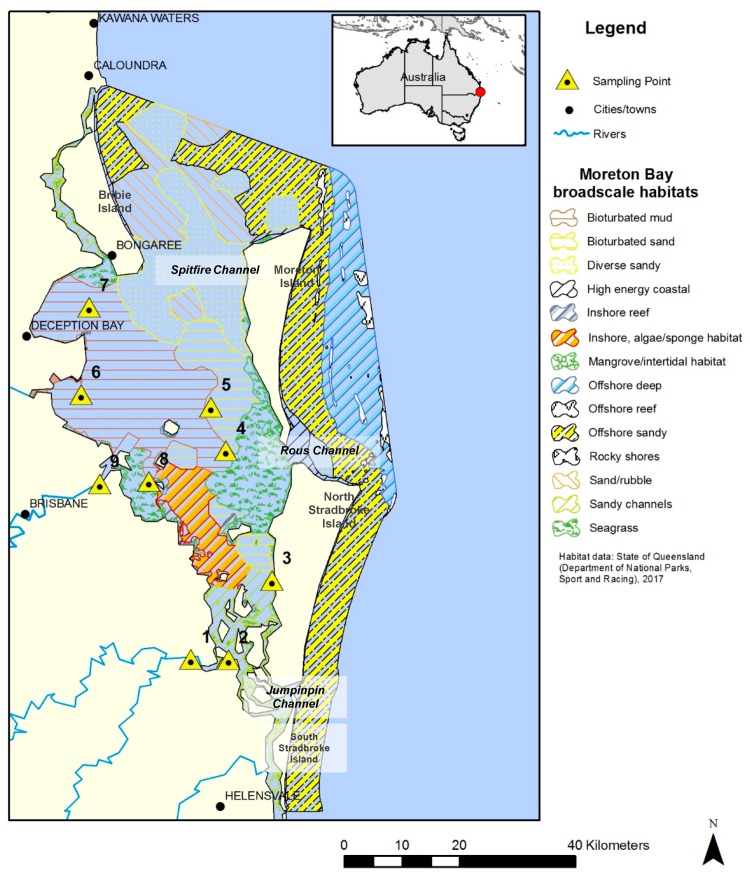
Map of Moreton Bay (South East Queensland, Australia), with its broad-scale marine habitats identified and sediment sampling sites annotated in yellow triangles.

One catchment/ecosystem of interest is Moreton Bay in South East Queensland, Australia (see Figure 1). Moreton Bay is a major sub-tropical shallow embayment. The bay is large (~1500 km^2^) and 125 km long, with a broad (35 km) opening at its widest west–east axis. This lagoon-like bay is surrounded by four large continental sand islands—Bribie, Moreton, North Stradbroke, and South Stradbroke [10]. The Bay has three openings to the Pacific Ocean. The northern passage between the Bribie and Moreton Islands is Spitfire Channel, which is the largest opening with the greatest oceanic exchange. Rous Channel, a smaller southern passage between the Moreton and North Stradbroke Islands, has a more restricted flow. Lastly, Jumpinpin Channel in the southern Bay between North and South Stradbroke Islands has the lowest degree of flushing [11]. The greater Moreton Bay catchment comprises 21,200 km^2^ of land, with four main rivers that drain into Moreton Bay: the Caboolture, Pine, Brisbane, Logan-Albert, and Pimpama Rivers. The river outflow includes effluent from riparian agriculture, treated sewage, and residential and industrial discharges, in addition to urban litter and pollution [10].

The Bay provides aquaculture, industrial, residential, and shipping services, making it vulnerable to environmental stresses. Being subtropical, Moreton Bay supports a diverse community of marine megafauna such as fish, migratory whales, corals, and subtropical species including sea turtles, dugongs, and Australian humpback dolphins [10]. The Bay also provides a variety of habitats for wildlife including extensive wetlands, shallow sandbanks, mudflats, mangrove forests, coral reefs, seagrass meadows, and myriad creeks, brooks, and rivulets [10]. Moreton Bay has been recently declared a marine park with recognition as a heritage site [12]. Historically, the Bay has been exploited for coral mining, sand mining, intense trawling, recreational fishing, and ecotourism [8]. In addition to increased urbanization in the adjacent catchment, such human activities adversely affect the ecology of this embayment. This subtropical region is predisposed to seasonal and mildly monsoonal rainfall during the wet season when freshwater runoff into the bay increases markedly, with occasional coastal flooding events [10]. Sediments, nutrients, and pollutants are mobilized and rapidly delivered to coastal waters [13]. The eastern and northern regions of the Bay are relatively well-flushed by oceanic exchange, whereas residence times in the middle, southern, and western Bay are generally longer (>40 days) [11]. As a result, riverine input has a greater influence on the southern and western Bay, where sediments are characterized by higher silt, organic carbon, and nutrient content [14]). Nutrients and chlorophyll in the western Bay have been reported to be 10-fold to 100-fold higher than in the eastern Bay [15]. Despite these large gradients, planktonic production remains the primary carbon input throughout the Bay, supplemented primarily by seagrass and mangrove production depending on local habitat type [16]. The Bay is thought to be nitrogen-limited, with nitrogen fixation accounting for 60% of the nitrogen input. Point source inputs, e.g., from wastewater treatment facilities and the Port of Brisbane, account for 85% of the phosphorus input and 25% of the nitrogen input [17]. It should be noted, however, that point source inputs have significantly reduced over the last 25 years with the move to tertiary wastewater treatment; as a result, agricultural runoff is the key concern. Industrial activities at the Port and shipping channel in the western Bay have also been linked to habitat degradation from toxic biocides derived from copper-based antifouling paints [18].

In recent times, omics-based techniques and their integration have been used to advance our understanding of microbial physiology. Kimes et al. [19] applied bacterial 16S rRNA gene sequencing and metabolite profiling techniques to characterize the microbial communities exposed to anthropogenic hydrocarbons and how their community composition and metabolic function impacted the bioremediation of oil spills. In a previous study, we used a similar approach to investigate surface water quality and characterize the bacterial population with respect to water contaminants in the Brisbane river [20]. A multi-omics-based ecological analysis of coastal marine sediments was also used to assess the impact of anthropogenic factors arising from point and non-point pollution sources at a multi-commodity marine port [21] and assess the impact of seasonal rainfall and urban pollution on marine sediment ecosystems [8]. In this study, we extend the application of bacterial 16S rRNA gene sequencing and metabolomics to assess the pressures of urban expansion, industrialization, and other factors associated with increased human activities on the Moreton Bay ecosystem. Specific stressors on the sediment microbial communities that were studied at various sediment depths include increased organic carbon inputs, nutrients and pollutants. This was done to present a baseline set of observations for the Moreton Bay ecosystem during the dry season and characterize the sediment community composition and function at different depths. Utilizing the principles of untargeted metabolomics, this also has the potential to generate new hypotheses for follow-up research activities within the studied catchment relating to ecosystem function [22]. Such an approach will provide insight into the influence of baseline residual contaminants within the sampled sediments, where the impact and influence of rainfall events and spike contamination events are removed. Together, this will enable a better understanding of the underlying envelope of conditions that define constructs of ecosystem health and resilience within Moreton Bay.

## 2. Materials and Methods

### 2.1. Sediment Sampling Sites

Marine sediment samples were collected in October 2015 from nine different sites surrounding four broad-scale habitats that occur within Moreton Bay. Figure 1 shows a map of Moreton Bay overlaid with the State of Queensland region habitat map. The sediment sampling sites are annotated with yellow triangles. The characteristics of each site in terms of the data collected in situ and sample metadata are presented in Table 1.

### 2.2. Sediment Core Collection

Sediment cores in the depth range 10–40 cm were collected using a gravity-hammer corer (Uwitec, Mondsee, Austria) in clear polycarbonate tubes (diameter 86 mm), as previously described [8]. Upon collection of sediment cores, the core tube was capped and immediately stored at −20 °C. In the laboratory, the sediment cores were divided into equal-depth horizons at 10-cm intervals in order to investigate the depth profile of bacterial communities and metabolites. This was achieved using a mechanical saw fitted with a stainless-steel blade, pre-cleaned with Pyroneg (Diversey Australia Pty Ltd., Smithsfield, NSW, Australia) and subsequently sprayed with 70% ethanol and MilliQ water between each cut. Each core depth sediment sample was thoroughly homogenised prior to subsampling after first removing large animal, shell, and plant material.

### 2.3. Physico-Chemical Analyses

All chemicals were of analytical grade or higher and were purchased from commercial sources unless otherwise stated.

#### 2.3.1. Sediment Grain Size Characterization

Particle size analysis was undertaken as previously described [8] and in accordance with the International Organization for Standardization (ISO) 14688–1: 2002 [23] and Australian Standard (AS) 4816.1–2002 [24]. Briefly, 40 g of oven dried marine sediment sample (60 °C for 48 h) from each site/depth horizon were sieved and a grain size classification expressed as a percentage was determined. The sieve stack consisted of stainless-steel sieves with mesh diameters of: 4.76, 2.36, 0.6, and 0.075 mm. Particle fractions were characterized as being gravel for sizes between 4.76 mm and 2.36 mm, coarse sand for sizes between 2.36 mm and 0.6 mm, fine sand for sizes between 0.6 mm and 0.075 mm, or silt for particle sizes less than 0.075 mm.

#### 2.3.2. Total Organic Carbon

Marine sediment samples (0.5 g) from each site were used for total carbon (TC) and total nitrogen (TN) measurement using a TruMac CN analyzer (LECO, Castle Hill, NSW). Total organic carbon (TOC) content was determined as per Beale et al. [8].

#### 2.3.3. Heavy Metal Analysis by Inductively Coupled Plasma Mass Spectrometry (ICP-MS)

Marine sediment samples (0.5 g) from each site/depth horizon were used for the analysis of trace heavy metals following EPA Method 3051A [25]. Trace heavy metals were extracted, in triplicate, using concentrated nitric acid (9 mL) by microwave-assisted digestion (Multiwave 3000, PerkinElmer Inc., Melbourne, VIC, Australia). Metals were analyzed using an Agilent 7700 × quadrupole-type ICP-MS (Agilent Technologies, Mulgrave, VIC, Australia), equipped with an Agilent ASX-520 auto sampler as per Beale et al. [8].

#### 2.3.4. Organic Pollutants Analysis by Liquid Chromatography–Mass Spectrometry (LC-MS/MS)

Organic pollutants were screened following the sample protocol previously published [8]. Sample extracts were reconstituted using 0.5 mL of mobile phase prior to LC-MS/MS analysis using an Agilent 6410 LC-QqQ (Agilent Technologies, Santa Clara, California, USA). An aliquot of 1.4 μL of reconstituted sample was injected onto a reversed-phase Agilent Zorbax Eclipse Plus C18 column (2.1 × 50 mm, 1.8 μm; Agilent Technologies, Santa Clara, CA, USA). The mobile phase A consisted of 0.1% formic acid in water and mobile phase B consisted of 0.1% formic acid in methanol. The column temperature was set to 30 °C and flow rate was set to 0.3 mL min^-1^. The instrument conditions were as per Beale et al. [8]. Briefly, samples were screened using an untargeted LC-MS/MS approach with two spiked standards coupled with a personal compound database library (PCDL) of known organic pollutants of concern as identified in the Australian and New Zealand Guidelines for Fresh and Marine Water Quality [26], with a library match criterion set at more than 80%. All identified and putative compounds were determined at a signal-to-noise threshold >50, and were subsequently normalized to 1% organic carbon, as per the standard protocol for marine sediment analysis from Beale et al. [8].

### 2.4. Bacterial 16S rRNA Gene Sequencing

DNA was extracted from approximately 5.0 g of sediment using a MO BIO PowerMax Soil DNA Isolation Kit (MO BIO, Carlsbad, CA, USA) as per Beale et al. [8]. Briefly, the extracted DNA samples were quantified using a NanoDrop spectrophotometer (ND-1000; NanoDrop Technology, Wilmington, DE, USA). The V5 and V6 regions of the 16S rRNA gene were amplified using the primer set described previously [27]. Amplicons from each sample were pooled in equal amounts. All samples were paired-end sequenced to a length of 250 nucleotides (nt) in each direction by the University of Minnesota Genomic Center (Minneapolis, MN), using version 3 chemistry on the HiSeq2500 platform. Raw data were deposited in the NCBI Sequence Read Archive under BioProject accession number SRP105345. Sequence processing was performed using mothur software (version 1.33.3, www.mothur.org) [28]. Sequences were first trimmed to 150 nt and paired-end joined using fastq-join [29]. Quality trimming was performed to remove sequences with average quality scores <35 over a window of 50 nt, homo-polymers >8 nt, ambiguous bases, or >2 mismatches to primer sequences. High-quality sequences were aligned against the SILVA database ver. 123 [30]. Sequences were further quality trimmed using a 2% pre-cluster [31], and chimera removal using UCHIME [32]. Assignment of operational taxonomic units (OTUs) was performed at 97% identity using the furthest-neighbor algorithm. Taxonomic assignments were made against the Ribosomal Database Project database ver. 14 [33]. For comparisons among sediment samples from sampling sites/depth, sequence reads for each sample were rarefied by random subsampling to 21,281 reads.

### 2.5. Community Metabolomics

Marine sediment samples were analyzed, in triplicate, using a modified untargeted Gas Chromatography–Mass Spectrometry (GC-MSD) metabolomics protocol previously described [22]. Briefly, the community metabolomics analysis was performed on an Agilent 6890A gas chromatograph (GC) oven coupled to a 5973A mass spectrometer detector (MSD)(Agilent Technologies, Mulgrave, VIC, Australia). The GC-MSD conditions were as stated previously [34,35,36]. Data acquisition and spectral analysis were performed as per Beale et al. [8] and according to the Metabolomics Standard Initiative (MSI) chemical analysis workgroup [37]. Procedural blanks (*n* = 7) were analyzed randomly throughout the sequence batch.

### 2.6. Statistical Analysis and Data Integration

The organic pollutant, trace metal, and physicochemical data were used to assess baseline contamination of the sampled sediment and the ecosystems from which they were collected. This was to account for any untoward bias in the subsequent bacterial 16S rRNA gene sequencing and metabolomics analysis. The sequence and metabolomics data were subjected to further statistical analysis using Principal Component Analysis (PCA) and Partial Least Square-Discriminant Analysis (PLS-DA) where PLS-DA was accomplished by finding successive orthogonal axes from the two or more datasets with maximum squared covariance and was subsequently used to identify the common relationships among the multiple datasets; these axes are derived by PCA. The data were first imported, matched by sample location identifiers (metadata), and log transformed to normalize the data. For this purpose, SIMCA 14.1 (MKS Data Analytics Solutions, Uméa, Sweden) was used. MetaboAnalyst 4.0 (Xia Lab, McGill University, Quebec, Canada) was used to determine significant metabolomics features [38] and METAGENassist (Wishart Research Group, University of Alberta, Alberta, Canada) was used to determine statistically significant bacterial sequence features [39] via volcano plots utilizing metabolite/bacterial 16S rRNA gene sequencing feature fold changes and the Benjamini–Hochberg adjusted *p*-values (*p* < 0.05). The taxonomic annotations of the sequences and automated taxonomic-to-phenotypic mapping were performed using METAGENassist. A fold change of <0.5 indicates a significant downward regulation; a fold change of >2.0 indicates a significant upward regulation [36]. For multiple group comparisons (≥3 groups), significant features were identified using one-way ANOVA statistical analyses (*p* < 0.05) using a post-hoc Fisher’s least significant difference method, as per Beale et al. [8].

## 3. Results

### 3.1. Physico-Chemical Analyses

#### 3.1.1. Sediment Grain Size Analysis

Marine sediments were characterized by particles ranging in size from gravel to silt (Supplementary Table S1). Overall, the sediment samples were consistent with previous studies [8] and were predominantly characterized as comprising combinations of coarse sand (1.7–51.3%) and fine sand (27.3–94.9%). A very high percentage of gravel was collected from site 2 (22% at depth 30 cm), site 6 (13.1–40.3%), site 7 (21.1% at depth 20 cm), site 8 (11.4–17.9%), and site 9 (13.5% at depth 20 cm and 20% at depth 30 cm). Sediments collected from other sites and/or core depths had a low percentage of gravel (<10%). Silt particles were very low in most samples (<5%) with a few exceptions such as site 3 (5.8% at depth 30 cm), site 4 (5.3%, 8.3 and 6.5% at depths of 10, 20, and 40 cm, respectively), site 8 (5.4% at depth 20 cm), and site 9 (8.5% at depth 10 cm).

#### 3.1.2. Total Organic Carbon Analysis

The total organic content is presented in Supplementary Table S2. The total carbon (TC) content ranged from 2.54 to 26.24 mg TC g^-1^ dry weight for sediments collected from all sites except site 8 (62.19% at depth 10 cm). The total organic carbon (TOC) ranged from 2.07 to 26.24 mg TOC g^-1^ dry weight. Inorganic carbon (IC) content was calculated to be <10% in most of the samples. While slightly higher inorganic content was found in site 5 (15.77–17.85 mg IC g^-1^ dry weight) and site 6 (12.64–13.81 mg IC g^-1^ dry weight), site 8 showed a considerably high inorganic content at depth 10 cm (55.09 mg IC g^-1^ dry weight). Total nitrogen (TN) content ranged from 0.17 to 1.95 mg TN g^-1^ dry weight. There appeared to be no correlation between sediment grain size and total organic carbon of the sampled sediments.

#### 3.1.3. Heavy Metals in Marine Sediments

Abiotic stressors such as heavy metals have a significant impact on natural flora and fauna. The concentrations for the metals quantified from each sampled site are presented in Supplementary Table S3. The table also presents the high and low trigger guidelines values as per the Australian and New Zealand Guidelines for Fresh and Marine Water Quality in sediments [26]. Overall, the metals analyzed were found to be below the instrument detection limits or the low trigger value for marine sediments, except for silver (site 9 at a depth of 40 cm) at 1.8 ± 0.3 mg kg^-1^; the low trigger value was set at 1.0 mg kg^-1^ and the high trigger value was set at 3.7 mg kg^-1^. As such, the impact of metals on the bacterial community structure and metabolism is considered uniform across all sampled sites.

#### 3.1.4. Organic Pollutant Analysis

A list of identified compounds and semi-quantitative concentrations based on two internal standards is provided in Supplementary Tables S4 and S5, respectively. As indicated in Supplementary Table S5, the organic pollutants were randomly distributed throughout the sampled sites and depth profile. However, it is apparent that site 3 at a depth of 20 cm was found to have elevated levels of the personal care products promethazine (an antihistamine) and DL-atenolol (a β-blocker), in addition to the pesticides carbamazepine and diethyltoluamide (DEET). Sites 4, 5, and 6 were also found to have elevated levels of promethazine, DL-atenolol, and DEET. While these values were considered low and below reportable levels of concern, it is indicative of an environment impacted by human activity. However, there were no clear trends of contamination or site-specific hot spots that would impact the microbial community structure and metabolism. As such, all the sampled sites were considered uniform from the perspective of organic contaminants.

### 3.2. Omics Analysis

Urban and industrial pollutants appeared to be minor or insignificant stressors at the sites sampled in this study within the context of the trigger values set in the Australian and New Zealand Guidelines for Fresh and Marine Water Quality [26]. The organic pollutant, trace metal and physicochemical data were used to assess baseline contamination of the sampled sediment and the ecosystems from which they were collected. This was to account for any bias in the subsequent 16S rRNA gene sequencing and metabolomics analysis if the sampled sites were above guideline values. Many of these data were collected as a discrete snapshot in time; without establishing an environmental gradient of “impact” or comparing against a pristine reference site, it is not possible to address the influence of human activities directly. It is acknowledged that the impacts of pollution on the microbial communities may be cumulative, episodic, site-specific or contributing to progressive deterioration across the entire ecosystem. This is a limitation of the current study. However, this work does provide an essential baseline and framework that will support further observations and experiments that can be used to define constructs of ecosystem health and resilience, not only in Moreton Bay but also other estuaries.

#### 3.2.1. Community Metabolomics

A total of 17,045 metabolite features (S/N ratio >10) were identified across all the sediment samples analyzed using GC-MS. Of these, 4468 were putatively identified using commercial metabolomics databases. This equates to an average of 356 ± 64 (± ½ range) metabolite features per sample. Of these, 93 ± 20 (± ½ range) were putatively identified. The internal standard, adonitol, was found to be within 13.2% residual standard deviations (RSD). Most metabolites identified were sugars, fatty acids, and amino acids.

Supplementary Figure S1 presents the PCA Score Scatter Plot and the Loading Scatter Plot for the metabolomics data. As illustrated in the PCA plots, there appeared to be clustering of sites, namely sites 6–9 were grouped away from the remaining sites (sites 1–5). This did not correlate with the site habitat classification or any of the physicochemical data but was most likely due to the influence of the Brisbane River. Sites 6–9 are strongly influenced by the Brisbane River, sites 4 and 5 are near the boundary of deposition of the Brisbane River, and sites 1–3 are outside the depositional area of the Brisbane River. Furthermore, sites 1–5 are most likely influenced by marine waters and nearby seagrass habitats. As such, additional PLS-DA (Supplementary Figure S2) and OPLS-DA (Supplementary Figure S3) models were used to investigate the sites and their differences.

Since biological datasets tend to vary significantly from sample to sample, a distance of observation (DModX) analysis was also used to identify, or eliminate, any outliers. DModX is the normalized observational distance between the variable set and X modal plane and is proportional to a variable’s RSD. DCrit (the critical value of DModX), derived from the F-distribution, calculates the size of observational area under analysis. The DModX plot (not shown) indicated that no samples exceeded the threshold for rejecting a sample. The threshold for a moderate outlier is considered when the sample DModX value is twice the DCrit at *p* = 0.05, which, in this instance, was 2.418 (DCrit = 1.209). This ultimately resulted in no data points being removed from the analysis.

**Figure 2:**
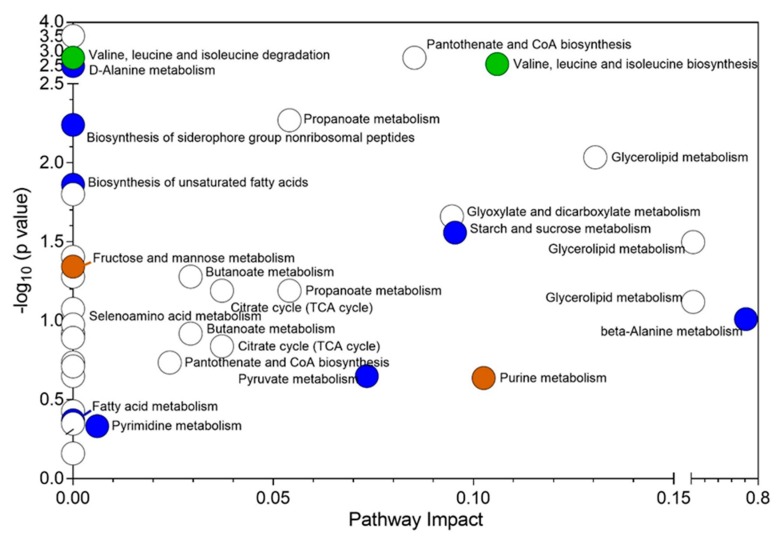
Metabolic pathway impact plot for Moreton Bay sediment samples based on site habitat. Note: the seagrass habitat samples were excluded from the analysis as they were under-represented in the sampling of the sites). Note: The Y-axis represents the log of the metabolite pathway significance (*p*-value) and the X-axis represents calculated pathway importance post pathway topology analysis (i.e., an importance score based on each metabolite’s betweenness and degree centrality). Blue, green, and brown circles represent pathways unique to river, sandy channel, and bioturbated mud, respectively.

The metabolomics data were subjected to multivariate analysis based on core depth. Interestingly, no significant metabolites were identified when core depth was the variable suggesting that the sediment bacterial community metabolism, irrespective of composition, is statistically unified at the depth sampled, irrespective of the taxonomic variance observed. This is not to say that the metabolites present did not vary through the core horizon, just that the metabolite abundance did not significantly change at the fold change threshold applied. However, the analysis of metabolomics from the perspective of habitat was found to be significant. Table 2 provides a list of identified significant metabolites with respect to habitat horizons. The unidentified significant metabolites are listed in Supplementary Table S6. The significant metabolites were then used to construct a metabolic pathway impact plot (Figure 2). Pathways related to metabolism of D-alanine, fatty acid, pyrimidine, pyruvate, -alanine, starch, and sucrose, and biosynthesis of unsaturated fatty acids and siderophore group non-ribosomal peptides were found to be unique to river samples. On the contrary, valine, leucine, and isoleucine degradation and biosynthesis were the only pathways unique to sandy channel sediments. Pathways related to purine, fructose, and mannose metabolism were unique to bioturbated mud sediments.

**Figure 3:**
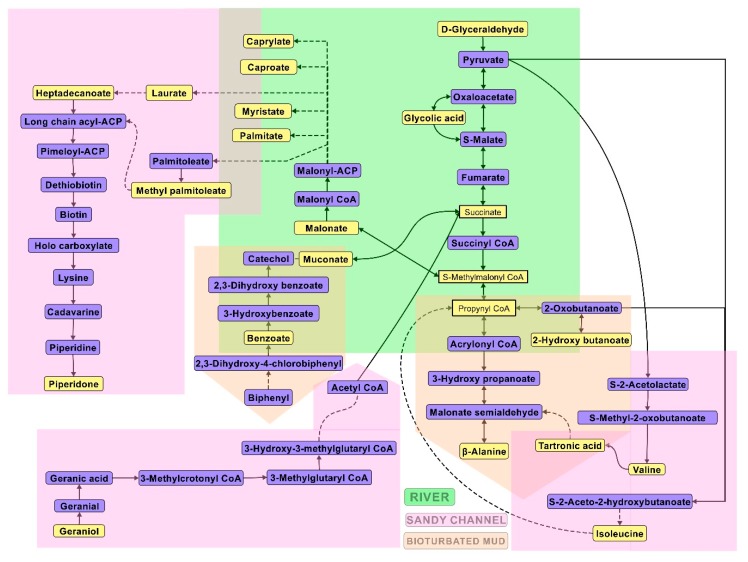
Important connecting metabolites expressed in various sediments. It was noted that the metabolic flux shifted downstream from river to sandy channels due to the differential microbial communities. Note: the metabolites highlighted with yellow boxes denote the primary interface metabolites with high fold change (FC) values, as detected by GC-MS.

By correlating the pathway impact and metabolite abundance, it was observed that there were numerous overlaps in the pathways expressed. This is best illustrated in the constructed metabolic pathway representation of the sampled sediments (Figure 3). It should be noted that, based on the collected metabolomics data, the metabolic outputs shifted downstream from river to sandy channels. This is most likely due to the differential microbial communities in these environments (presented in the proceeding sections).

It was observed that the river ecosystem had a significant influence on the overall community metabolic activity downstream. For example, glyceraldehyde, succinic acid, malonic acid, and β-alanine were the most abundant metabolites. However, due to lower dissolved oxygen levels in the river system, it was observed that riverine metabolomics was dominated by fermentation and anoxic pathways. The glycolysis pathway via pyruvate metabolism resulted in the biosynthesis of succinic acid, bypassing the citrate cycle. Succinate was in turn further fermented to malonic acid, which further acted as a precursor to β-alanine synthesis via propanoate and β-alanine metabolism pathways and fatty acid biosynthesis.

Some of the β-alanine metabolism seemed to be transferred from the river to bioturbated mud ecosystem. A similar trend was seen with respect to the degradation of polychlorinated biphenyls (PCBs). PCBs from the river system were observed to be degraded by the microbial community in bioturbated mud via the benzoate degradation pathway to form muconic acid, which in-turn completed a feedback loop into possible fatty acid biosynthesis or β-alanine metabolism via succinate as an intermediate.

The glycolysis components flushed from the riverine and bioturbated mud ecosystems were re-located into sandy channels. One of the most dominant pathways observed was valine, leucine and isoleucine biosynthesis which appeared to integrate the outputs of propanoate (bioturbated mud) and pyruvate metabolisms (river). On the other hand, monoterpenoids such as geraniol which could not be degraded in river and bioturbated mud were slowly degraded by communities in sandy channels. The metabolism preceded via geraniol and leucine degradation pathways to feed back to succinate. Fatty acids produced in the riverine system were further processed to generate long-chain fatty acids and piperidine metabolism.

#### 3.2.2. Bacterial 16S rRNA Gene Sequencing

**Figure 4:**
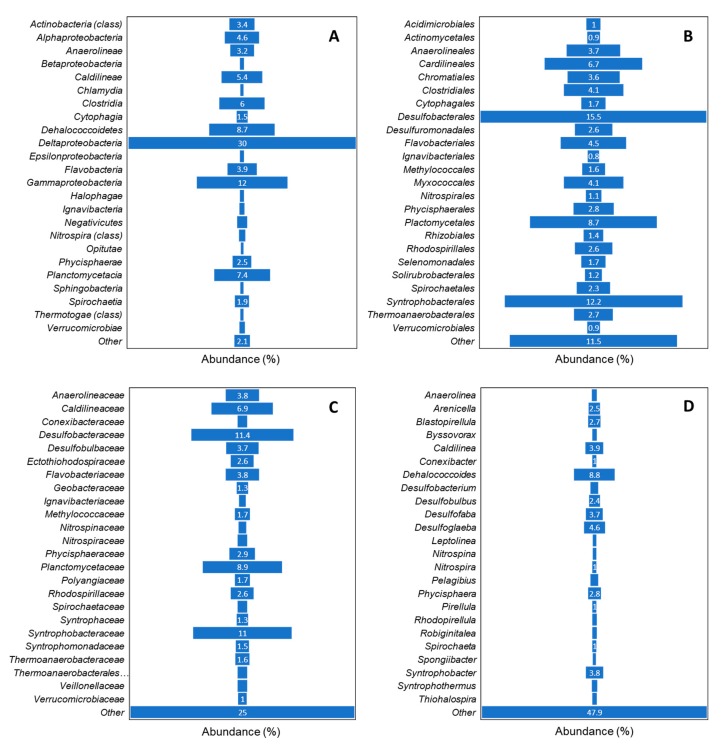
Taxonomic classification of bacterial communities in Moreton Bay sediment samples for cohort (**A**) Class, (**B**) Order, (**C**) Family and (**D**) Genus.

Figure 4 provides a summary of the bacterial taxonomic data for sediment from all nine sampled sites across habitats. The most prominent class of bacteria found in the sediment samples was *Deltaproteobacteria* followed by *Gammaproteobacteria*, *Dehalococcoidetes*, *Planctomycetacia*, *Clostridia*, *Caldilineae*, *Alphaproteobacteria*, *Flavobacteriia*, *Actinobacteria* and *Anaerolineae*. The bacterial communities observed in the current study indicated the members that require no oxygen (either anaerobic or anoxygenic) and moderate temperatures (i.e., mesophilic) for growth. This was further supported by phenotypic analysis based on oxygen and temperature requirements and metabolic functions exhibited by these bacterial communities. Bacteria in these samples most likely carried out one of the five major metabolic functions: sulfur reduction, dehalogenation, ammonia oxidation, xylan degradation, and nitrogen reduction.

### 3.3. Variation between Core Depths and Habitat

#### 3.3.1. Based on Taxonomy

**Figure 5:**
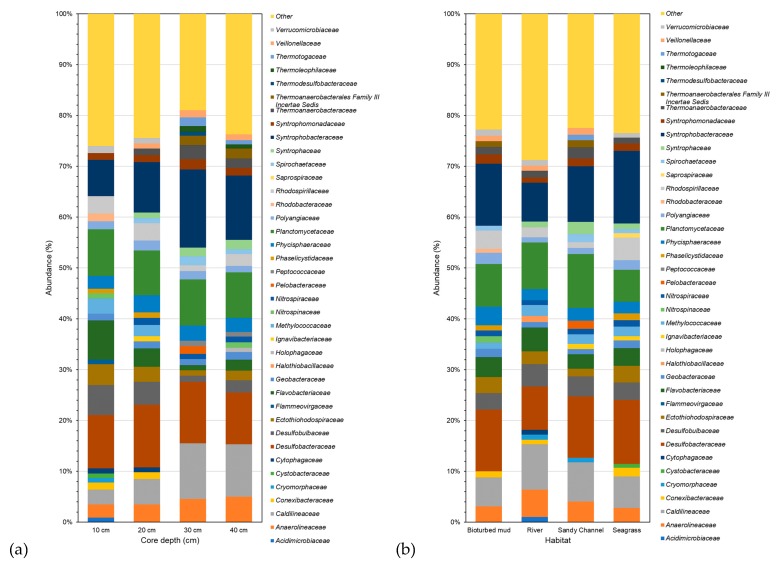
Bacterial classification among (**a**) core depth sampled sites by taxonomy for the cohort family and (**b**) habitat sampled sites by taxonomy for the cohort family.

Phylogenetic classification revealed differences among samples from various core depths at sediment sites. Figure 5 illustrates the average bacterial phylogenetic characterization as determined in METAGENassist for each cohort family based on sediment core depth (Figure 5a) and habitat sampled (Figure 5b). The bacterial differences for each cohort class, order, and genus are presented in Supplementary Figure S4. *Desulfobacteraceae* dominates the sediment samples at core depth of 10 cm and 20 cm, while *Syntrophobacteraceae* dominates at core depth of 30 cm and 40 cm (Figure 5a). These were followed by *Planctomycetaceae* at 10 cm, *Syntrophobacteraceae* at 20 cm, *Desulfobacteraceae* at 30 cm, and *Caldilineaceae* at 40 cm (Figure 5a). Bacteria from the class *Deltaproteobacteria* dominated in all samples from various core depths. This was followed by bacteria in class *Gammaproteobacteria* in samples from core depths of 10 cm and 20 cm and class *Dehalococcoidetes* in samples at 30 cm and 40 cm (Supplementary Figures S4 and S5).

Within the bacterial community, *Deltaproteobacteria* dominated all four habitats, followed by *Gammaproteobacteria* in bioturbated mud, river, and seagrass and *Dehalococcoidetes* in sandy channel. The results in Figure 5b suggest bacteria from the *Anaerolineaceae, Caldilineaceae, Desulfobacteraceae, Desulfobulbaceae, Ectothiorhodospiraceae, Flavobacteriaceae, Geobacteraceae, Methylococcaceae, Nitrospiraceae, Phycisphaeraceae, Planctomycetaceae, Polyangiaceae, Rhodospirillaceae, Syntrophobacteraceae, Syntrophomonadaceae* and *Thermoanaerobacteraceae* families were detected in samples from all habitats. It is worth noting that sediments from the bioturbated mud site are abundant in bacteria from the *Syntrophobacteraceae* and *Desulfobacteraceae* families. *Caldilineaceae* and *Planctomycetaceae* dominate the river sediments. Whilst *Desulfobacteraceae* dominated the bacterial community in the sandy channel sediment samples, *Syntrophobacteraceae* contributed to the highest percentage of bacteria in seagrass sediments. Although *Desulfobacteraceae* and *Syntrophobacteraceae* were found in samples collected from all sites, they were significantly less abundant in river sediments. Similarly, *Planctomycetaceae* was less abundant in seagrass as compared to other sediment sites. *Rhodospirillaceae* was found to be significantly higher in bioturbated mud and seagrass. Bacteria from *Nitrospinaceae* and *Rhodobacteraceae* were identified in the bioturbated mud samples only, albeit at low abundances. Similarly, low abundances of bacteria from the *Acidimicrobiaceae, Cytophagaceae* and *Halothiobacillaceae* families, bacteria from the *Pelobacteraceae* and *Thermotogaceae* families, and bacteria from the *Cystobacteraceae* and *Saprospiraceae* families were identified in the river, sandy channel and seagrass sediment samples, respectively. While it would be advantageous to propose specific indicator species that represent community health or stress against a background of community variation in each habitat, more work is needed to assess these communities over time in order to establish indicators that are robust, resilient, and reliable.

#### 3.3.2. Based on Phenotype

The sediment samples were also classified based on their phenotype. Bacteria at core depth of 10 cm were found to be aerobic (26.4%) and those at other core depths were found to be anaerobic (29.7% at depth 20 cm, 28.3% at depth 30 cm, and 34.9% at depth 40 cm). Based on temperature requirements, bacteria in all samples were found to be significantly more abundant in mesophilic conditions (30% at depth 10 cm, 26.9% at depth 20 cm, 16.9% at depth 30 cm, and 20.9% at depth 40 cm). Thermophilic bacteria were significantly higher in deeper sediments (14.8% at depth 30 cm and 14.4% at depth 40 cm as compared to 8.6% and 10.9% at depths 10 cm and 20 cm, respectively).

A very large percentage (70–78%) of bacteria obtained their energy from unknown sources. Most of the remaining bacteria were autotrophic (7.5% at depth 10 cm and 7.8% at depth 20 cm) or organotrophic (6.8% at depth 30 cm and 7.2% at depth 40 cm). Sulfur reduction was found to be the most dominant metabolic function in all samples at various core depths (28.6% at depth 10 cm, 28.1% at depth 20 cm, 17.6% at depth 30 cm, and 21% at depth 40 cm). This was followed by dehalogenation (23.9% at depth 10 cm, 23.1% at depth 20 cm, 15.5% at depth 30 cm, and 18% at depth 40 cm) and ammonia oxidation (22.7% at depth 10 cm, 20.6% at depth 20 cm, 12.5% at depth 30 cm, and 4.9% at depth 40 cm). Other prominent metabolic functions identified were xylan degradation (8–15%), sulfide oxidation (6–9%), nitrite reduction (8–14%), nitrogen fixation (7.5–10%), aromatic hydrocarbon degradation (4.5–6.5%), and chitin degradation (2–4.5%).

The sediment samples from the four habitats were also classified based on their phenotype. Anaerobic bacteria dominated the sediment samples from all habitats (29.1% in bioturbated mud, 31.1% in river, 34.5% in sandy channel, and 32.1% in seagrass). Sediment samples from various habitats were found be abundant in mesophilic bacteria (bioturbated mud: 24.4%, river: 25.3%, sandy channel: 23.2%, and seagrass: 22.9%).

Whilst a significant bacterial population (72–75%) obtained their energy from unknown sources, many of the remaining bacteria were autotrophic in bioturbated mud (7.4%), sandy channel (6.4%) and seagrass (7.3%) or organotrophic in river (7.3%). Sulfur reduction was found to be the most dominant metabolic function in all samples from different habitats (24.8% in bioturbated mud, 23.7% in river, 22.9% in sandy channel, and 28.3% in seagrass). This was followed by dehalogenation (20.4% in bioturbated mud, 20.5% in river, 19.7% in sandy channel, and 23.7% in seagrass) and ammonia oxidation (18.8% in bioturbated mud, 17.5% in river, 17% in sandy channel, and 18.9% in seagrass). Other prominent metabolic functions identified were xylan degradation (11–15%), sulfide oxidation (6.5–8%), nitrite reduction (10–12%), nitrogen fixation (8–10%), aromatic hydrocarbon degradation (5–7%), and chitin degradation (3–3.5%).

## 4. Discussion

The Australian and New Zealand Guidelines for Fresh and Marine Water Quality provide a framework to classify sampling sites based on their ecosystem condition [26]. In this study, based on the Guidelines, Moreton Bay is considered a moderately disturbed ecosystem of high ecological value. As such, the physical and chemical characteristics are assessed against the sub-regional guidelines for Moreton Bay [40]. Sites 1–3 and 6–9 are considered enclosed coastal waters or lower estuary sites, while sites 4 and 5 are considered open coastal estuary waters. Sites 1–5 and 8 are also noted to be of high ecological value marine/estuarine waters.

As previously stated, Moreton Bay receives water from a coastal and hinterland area of ~22,000 km^2^. This includes rainwater runoff, effluent from riparian agriculture, treated sewage, residential and industrial outflow, and urban litter. Such modifications have led to deterioration of the bay water and sediment quality in terms of increased nutrient, heavy metals and contaminant fluxes. These sediments exposed to urban pollutants may have long-term human and environmental implications. Nutrients, such as nitrogen and phosphorus, are essential for plant and animal growth and nourishment. Over-abundance of these nutrients in water can result in over-stimulation of growth of nuisance aquatic plants and algae. The total nitrogen concentration was above the trigger value of 150 μg N L^-1^ for all samples. The total organic content (TOC), in addition to sediment grain size, is known to influence the adsorption capacity of sediments and also the binding of organic pollutants to the sediments [41]. The combined high percentage (58–100%) of coarse and fine sand found in the sediments with a high TOC concentration of (2.07–6.24 mg TOC g^-1^ dry weight) may increase the adsorption capacity (larger surface area) of the sediments and result in considerably higher binding of organic pollutants (hydrophobicity of TOC). No trends, however, were observed between samples. The TOC data were used to normalize the organic pollutant data as per standard practice. Heavy metal pollution of marine sediments is typically associated with human activities such as mining or discharges from manufacturing industries [41]. High concentrations of metals in sediments can potentially have toxic effects on resident aquatic ecosystem and can make fish, crustaceans, and bivalves unsuitable for human consumption [42]. Coates-Marnane et al. [12] reported trace metal (lead, zinc and copper) deposits in sediment core samples collected in the Moreton Bay region. Heavy metals, albeit below trigger values, were found in our previous studies at all sites sampled in this region [8]. All organic pollutants except pindolol were found in most of the samples from various sampling sites. A few samples sites reported organic pollutants above the threshold for chemical stressors at the 25% probability of biological effects (80–100 μg g^-1^ OC dry weight) [43]. Organic pollutants reported above the threshold were penicillin V and DL-atenolol for site 1; penicillin V, diphenhydramine, promethazine, carbamazepine, N,N-diethyl-meta-toluamide, and DL-atenolol for site 3; promethazine and DL-atenolol for site 4; promethazine, N,N-diethyl-meta-toluamide, DL-atenolol for sites 5 and 6; and penicillin V, promethazine, and carbamazepine for site 9. No organic pollutants were reported above the threshold for sites 2, 7, and 8.

Large populations of sulfate and sulfur metabolizing bacterial communities (*Deltaproteobacteria*, 30%; *Gammaproteobacteria*, 12%) and organohalide respiring bacteria (*Dehalococcoidetes*, 8.7%) were detected in collected sediment samples. Members of the families *Desulfobacteraceae* (11.4%) and *Syntrophobacteraceae* (11%) were the most important taxonomic groups in terms of abundance and activity (in terms of metabolism). These results align well with the previous findings [44]. Recent estimates suggest that 29% of organic matter deposited to the seafloor is remineralized by sulfate-reducing microorganisms (SRMs) such as *Deltaproteobacteria* [45]. SRMs cooperatively degrade organic matter and, as such, are conventionally regarded as the terminal components of anaerobic food webs [46]. Overall, the SRMs were observed to work in commensal relationship with methanotrophs and methylotrophs, especially towards the lower end of bioturbated muds and overlapping zones of these muds and sandy channels. This was especially observed in the degradation pathways of aromatics and polychlorinated pollutants washed off from the riverine system. While the methylotrophs were active in the river sediments, especially at 20-cm depth, SRMs were more active in the bioturbated mud and sandy channels. The methylotrophs were observed to be involved in the degradation of PCBs and pyruvate metabolism; the products of these metabolic pathways were further processed in bioturbated muds (further elaborated below).

Methanotrophs such as *Methylococcaceae* formed a considerable part of bioturbated mud and sandy channel communities. SRMs such as *Desulfovibrionaceae* were observed to utilize the products of methanotrophs to generate CO_2_ via sulfate acceptors via non-stoichiometric oxidation processes. Although the Bay is classified as one of the shallower water bodies, these demineralization activities in sandy channels and bioturbated muds showed similarities to studies of deeper water bodies [47,48]. Organohalide-respiring microorganisms (ORMs) such as *Dehalococcoidetes* reduce chlorinated compounds into less toxic or harmless products through the anaerobic reductive dechlorination in the presence of an electron donor such as direct H_2_ or fermentable organic substrates [49].

Biogeochemical zonation in sediments was not measured in this study, as it was deemed out of scope for this research and relevant data from prior studies are limited. Moriarty et al. [50] found that 90% of the sulfate reduction occurred in the top 5 cm of two cores from seagrass habitats in Moreton Bay. The authors also found that methanogenesis accounted for <2% of sediment carbon flow through the microbial population, which was consistent with the view that methanogenesis is low in Moreton Bay sediment. We expect that this short length scale of zonation reported in seagrass sediments by Moriarty et al. [50] could vary between different habitat types, but there is a paucity of direct geochemical process measurements to validate this speculation. Alternatively, we examine the biogeochemical role of sediment bacterial populations based on metabolic phenotype (e.g., Figure 4).

Sediment accumulation in Moreton Bay is driven primarily by episodic delivery of riverine sediment loads during major floods, which form extensive deposits of bioturbated mud and sand in the eastern Bay (Figure 1). Prior studies have reported high sediment accumulation rates in these depositional regions (>1 cm yr^-1^, [13,51]) and shown that sediment at the depths sampled in this study (0–40 cm) can be attributed to anthropogenic activity in the catchment over the past 100 years. Depths of active bioturbation are unknown, but analysis of geochemical tracers in mud cores by Coates-Marnane et al. [13] indicate recent mixing of sediments to depths >40 cm. Morelli and Gasparon [12] found less mixing in cores from shallow and intertidal environments in Moreton Bay; cores near seagrass-dominated regions showed the lowest disturbance, with mixing confined to the top 10 cm.

Table 3 provides a summary of the bacterial 16S sequencing data based on unique features (out of top 25 features) per class, order, family and genus for each core depth and for each habitat. More unique features at various core depths were observed when examined at family and genus level. The taxonomic classification revealed that the sediment samples at all core depths and from different habitat horizons had ≤2 unique features based on class and order.

The unique features identified at the family level for sediment samples were *Acidimicrobiaceae, Cryomorphaceae, Cystobacteraceae, Flammeovirgaceae* and *Rhodobacteraceae* for core depth 10 cm; *Ignavibacteriaceae* for core depth 20 cm; *Pelobacteraceae* and *Thermodesulfobacteraceae* for core depth 30 cm; and *Holophagaceae* for core depth 40 cm. At the genus level, the unique features were *Actibacter, Desulfococcus, Eudorea,* and *Methylonatrum* for core depth 10 cm; *Phaselicystis* for core depth 20 cm; *Desulfatiferula, Fervidicola, Isosphaera, Pelobacter, Thermacetogenium,* and *Thermococcoides* for core depth 30 cm; *Smithelia* for core depth 40 cm.

At the family level, *Acidimicrobiaceae, Cytophagaceae* and *Halothiobacillaceae* were identified as the unique features in river sediments. The unique features in sandy channel sediments were *Pelobacteraceae* and *Thermotogaceae*; in bioturbated mud they were *Nitrospinaceae* and *Rhodobacteraceae* and in seagrass they were *Cystobacteraceae* and *Saprospiraceae*. At the genus level, no unique features were identified in seagrass. The unique features were *Actibacter* and *Planctomyces* for river sediments; *Desulfobacca, Ignavibacterium, Pelobacter, Smithelia,* and *Spirochaeta* for sandy channel, and *Desulfatiferula* and *Nitrospina* for bioturbated mud.

The bacterial 16S rRNA gene sequencing datasets were collated and analyzed using multivariate statistics in order to assess Moreton Bay sites from the perspective of depth-profiling. As such, the sites were pooled and core depth was used as a factor for investigation. The main points of difference in bacterial families identified between sampled depths using multivariate statistics are detailed in Table 4. It should be noted that members of family *Desulfobulbaceae* that belong to SRMs and the family *Caldilineaceae* that belong to ORMs were identified as significant bacterial features distinguishing between sediments samples from different core depths. These observations align well with the predicted metabolic functions based on the bacterial 16S rRNA gene sequencing data. Dehalogenation and sulfate reduction were found to be two most significant metabolic functions performed by the bacterial community. Table 5 presents a summary of predicted phenotypic classification using multivariate statistics. The metabolomics data for depth-profiling was assessed using multivariate statistics. The absence of any significant metabolites indicates that the microbial metabolome is largely similar within the sediments sampled at various core depths, that is, the abundance of metabolites either did not meet the fold change criteria or the *p*-value threshold. Microbial metabolomes from deeper sediments suggested differences between microbial communities based on their metabolic functions.

Aerobic organisms were found to be significantly more abundant within top 20 cm sediments. Microbes in this region are known to source their energy from methane and other inorganic compounds containing sulfur, nitrogen or iron. Microbes at depths of 30 cm and 40 cm were anaerobic in nature and sourced their energy from both organic and inorganic compounds. Aerobic bacteria such as *Rhizobiaceae*, *Conexibacteraceae* and *Flavobacteriaceae* were dominant in the top 10 cm of sediment. Anaerobic bacteria such as *Methylococcaceae* and *Desulfuromonadaceae* were also found to dominate. Methanotroph bacteria (*Methylococcaceae*) were the most abundant species at depths of 10 cm and 20 cm. Nitrogen fixing, diazotrophic, aerobic bacteria such as *Rhizobiaceae* were also identified within the top layer. Anaerobic bacteria such as *Thermoactinomycetaceae* were dominant at depths of 30 cm and 40 cm. SRMs such as *Ectothiorhodospiraceae, Granulosicoccaceae* and *Chromatiaceae* often found in marine environments were also identified in the sediments from Moreton Bay. The halotolerant *Oceanospirillaceae*, often found in marine environments, were also detected in sediments.

*Methylcoccaceae* were some of the differential communities in the marine ecosystem. The metabolomics output suggested that certain family members utilized the high nitrogenous substrates present in sediments (See Table 1) to feed it back to glycolysis via the pentose phosphate pathway to pyruvate metabolism, with additional feeding from starch metabolism by other communities. Recent studies have indicated that the salt tolerant members of *Methylcoccaceae* such as *Methylococcus capsulatus* have the ability to process nitrogenous substrates via the Benson-Calvin cycle in the presence of methane. This can also be inferred from the considerable upregulation of palmitic acid, which is known to be a major metabolite of this family and other methanotrophs [52]. Similarly, *Geobacteraceae* activity suggested their role in both the production of isoleucine and valine by recruiting 2-hydroxy butanoate from propanoate metabolism, with pyruvic acid as a precursor, as demonstrated in an earlier study [53]. The metabolic activity also suggested a conversion by Fe (III) mobilization in the overlapping regions between bioturbated mud and sandy channels. Similarly, in the bioturbated mud, these bacteria appeared to use fumarate as an electron acceptor to degrade the benzoic acid (the product of PCB degradation in the riverine ecosystem) via muconic acid intermediate to pyruvate metabolism. This ability has been suggested by Röling [54], where these bacteria work in commensalism with sulfate-reducing bacteria to degrade organic pollutants such as PCBs and similar polychlorinated compounds resulting from human activities.

A high β-alanine content in the riverine sediments indicated the presence of high degradation of organic matter in sediments, especially at shallow depths. Generally, in marine environments, β-alanine is formed as a degradation product of aspartic acid and a high Asp/ β-alanine ratio indicates the presence of fresh organic matter [55,56]. However, in the current study, it appears that a high perturbation in riverine and bioturbated sediments, combined with high nitrogen content, resulted in considerably high biosynthesis of β-alanine through other pathways as well. Similarly, a high β-alanine content (very low Asp/ β-alanine ratio) in this study also indicates a considerable decrease in the amount of fresh water (with respect to saline water) among the sediment samples. These outputs also indicated a high upregulation of β-alanine-pyruvate transaminase amongst the microbial communities in the river and bioturbated mud ecosystems. This output is in line with the previously studied geo-biochemical pathways occurring due to the polluting activities in the estuarine sediments [56,57].

The bacterial 16S rRNA gene sequencing datasets were also assessed from the perspective of habitat horizons. The main points of difference in terms of taxonomy (bacterial families) and phenotype identified between sampled horizons using multivariate statistics are detailed in Table 6 and Table 7**,** respectively.

Bacteria such as *Sphingomonadaceae* that degrade aromatic organics were abundant in the river habitat. Bacteria with diverse characteristics were found to dominate in the river habitat such as thermophilic *Hydrogenophilaceae*, diazotrophic *Oxalobacteraceae* and the halotolerant mesophilic chemolithotrophic bacteria *Halothiobacillaceae*. It is worth noting that bacteria from the family *Neisseriaceae* were also identified in the river habitat. Members of this family are known to cause gonorrhea and meningitis. Streptomycin-producing bacteria such as *Streptomycetaceae* were also found in the river habitat.

Organisms in the river habitat were more likely to be photoheterotrophs which use light energy but rely on carbohydrates, fatty acids and alcohols as a carbon source. Most microbes in the river habitat reduce nitrates to release nitrogen into the atmosphere and oxidize sulfur. Nitrogen-fixing bacteria were dominant in the sandy channel habitat. Lignin-degrading bacteria were most likely to be abundant in the bioturbated mud.

The comparison of bacterial 16S rRNA gene sequencing and metabolomics datasets suggest increased anoxygenic conditions in the sediments. This was most marked in the bioturbated mud site sediments. These results indicate a decrease in faunal activity in bioturbated mud sediments (Figure 2) and may result in decreased oxygen supply. Similar results for sediments from Moreton Bay were reported by our group during the dry season [8]. This possibly resulted in an increase in the population of SRM such as *Desulfobulbaceae*, *Desulfovibrionaceae* and other similar species. The increased population of SRM may have changed the metabolite constitution of those sediments. This is indicated by an increased fatty acid metabolism. A putative indicator of increased fatty acid metabolism was palmitic acid (Table 2). The role of these bacteria in β-oxidation has been reported earlier [8,58,59] and our results are in alignment with these observations. An increased population of SRM also has an impact on sugar and sucrose metabolism [8]. This was particularly evident in river sediment samples by the presence of trehalose and maltose (Table 2).

The metabolomics dataset was subjected to pathway impact analysis (Figure 2) and pathway analysis (Figure 3). Based on the statistically significant metabolites identified using Fisher’s LSD analysis (Table 2), sandy channel and river habitat sites were found to be very active. Supplementary Table S7 presents the predicted metabolic pathways based on significant metabolites identified from sediment samples collected from various habitat sites as determined by the Fisher’s LSD analysis. Glycerolipid metabolism was the only common pathway observed in all habitat sediment sites. The presence of metabolites such as inosinic acid and deoxycytidine in bioturbated mud and river sediment samples, respectively, suggests that a considerable amount of biotransformation related to purine and pyrimidine metabolism occurred at those sites. The bacterial population present at the mouths of the rivers, most likely, were able to divert the pyrimidine metabolism via pantothenate and/or β-alanine intermediate pathways.

Bioturbated mud sediment samples exhibited fructose and mannose metabolism indicated by the presence of mannitol. We observed succinic acid in river and sandy channel sediments, which was indicative of propanoate metabolism, butanoate metabolism, citrate cycle, and glyoxylate and dicarboxylate metabolism. Detection of glycolic acid in river sediment samples also indicated glyoxylate and dicarboxylate metabolism. The detection of valine and α-ketoisovaleric acid, especially in the sandy channel habitat samples, could be attributed to metabolism of complex amino acids such as valine, leucine and isoleucine possibly via propanoate metabolism, butanoate metabolism, pyruvate metabolism, citrate cycle, or β-alanine pathways. Interestingly, 2-pyrocatechuic acid was observed in river sediment samples. This is indicative of a siderophore biosynthesis pathway. Siderophores are ferric iron chelators secreted by microorganisms for survival in low-iron environments, which was supported by the observed low concentrations of iron in the sampled sediments. Other notable metabolic pathways found to be unique in river habitat were D-alanine metabolism, pyruvate metabolism and selenoamino acid metabolism.

## 5. Conclusions

Habitat type was the major control on bacterial community composition when Moreton Bay was considered as a large estuary with diverse habitats and moderate human activity. The impact of other stressors such as heavy metals and organic pollutants appeared to be insignificant relative to habitat type based on guideline trigger vales. However, it is noted that such contaminants can have an accumulation affect and the discrete snapshot analysis presented here only provides a baseline for future research to be compared against. With that in mind, the differences in bacterial communities between habitats were expected due to different energy sources and biogeochemical processes. For example, sulfur reduction was highest in seagrass and bioturbated muds where oxygen is actively transported to deeper sediments. Similarly, organotrophic bacteria and ammonia-oxidizing bacteria were most abundant in organic-rich muds and least abundant in sandy channel sediments. Despite these apparent trends, most bacteria obtained their energy from unknown sources. As such, more work is needed to characterize the trophic/metabolic links within these marine sediments, i.e., autotroph → heterotroph 1 → heterotroph 2 → heterotroph 3. This would be best served by a comprehensive multi-omics (metabolomics, proteomics and metagenomics) study, utilizing metabolite analyses via both LC and GC platforms in order to better represent the metabolome. Furthermore, undertaking a targeted analysis of key expressed pathways that encompass nitrite reduction, dehalogenation, ammonia oxidation and xylan degradation would be appropriate.

Gradients in bacterial composition with depth were less distinct than typically conceptualized in biogeochemical zonation models, i.e., where the oxidant with the greatest free energy is utilized first and entirely depleted before the next most efficient oxidant is used (e.g., Burdige [60]). The absence of strong community gradients may imply a more dynamic nature of sediments. For example, chemical distributions may reflect an immediate but potentially unstable state, while biological distributions reflect more stable conditions over longer timescales. Alternatively, it may be more likely that the chemical gradients are stable, and the facultative bacteria actively move between different redox conditions. Again, more research is needed to establish such links.

## Figures and Tables

**Table 1:** Summary of characteristics of each sampling site and the discrete measurements collected in-situ (average over 5 min, continuous sampling).

Site	Habitat	Water Column Depth (m)	Sediment Core Depth (cm)	Water Temp. (°C)	Salinity	pH	DO (%)	NTU	Phosphorus as P (mg L^-1^)		Nitrogen as N (mg L^-1^)				GPS Coordinates	
									TDP	TP	NH_4_/NH_3_	NOx	TDN	TN	Latitude	Longitude
1	River	3	40	24.7	30.4	7.9	96.5	7.9	0.093	0.097	0.150	0.130	0.670	0.400	S 27 41.748	E 153 16.938
2	Sandy channel	3	40	24.2	31.7	7.9	92.8	5.4	0.030	0.040	0.056	0.024	0.280	0.180	S 27 41.784	E 153 20.454
3	Sandy channel	5	30	23.6	35.8	8.1	102.8	1.3	0.018	0.025	0.100	0.012	0.320	0.490	S 27 34.416	E 153 24.516
4	Bioturbated mud	25	40	22.5	35.8	8.1	101.2	<0.1	0.012	0.050	0.015	0.003	0.150	0.370	S 27 22.278	E 153 20.214
5	Bioturbated mud	15	40	22.4	35.8	8.1	101.6	<0.1	NA	NA	NA	NA	NA	NA	S 27 18.210	E 153 18.822
6	Bioturbated mud	5	40	23.6	35.2	8.0	96.1	3.2	0.063	0.039	0.310	0.037	0.960	0.290	S 27 17.034	E 153 06.666
7	Bioturbated mud	5	30	26.3	35.7	8.1	110.4	4.4	0.016	0.037	0.014	0.008	0.230	0.360	S 27 09.162	E 153 02.136
8	Seagrass	8	20	24.1	36.0	8.2	101.5	1.7	0.025	0.025	0.099	0.012	0.290	0.460	S 27 25.158	E 153 13.014
9	River	3	40	22.7	32.6	8.0	90.9	5.5	0.091	0.097	0.130	0.140	0.550	0.480	S 27 25.374	E 153 08.400

DO: dissolved oxygen; NTU: turbidity; TDP: total dissolved phosphorus; TP: total phosphorus; NH_4_/NH_3_: nitrogen in the form of ammonia; NO_x_: nitrogen in the form of oxide; TDN: total dissolved nitrogen; TN: total suspended nitrogen; NA: data not available.

**Table 2:** Identified significant metabolites from the different marine sediment habitats sampled from Moreton bay.

Metabolite	F-Value	*p*-Value	FDR	Fisher’s LSD
Malonic acid	33.646	1.50E-11	1.31E-09	Bioturbated mud > River; Bioturbated mud > Sandy channel
L-Canavanine	33.509	1.62E-11	1.31E-09	Sandy channel > Bioturbated mud; Sandy channel > River
L-Valine	33.509	1.62E-11	1.31E-09	Sandy channel > Bioturbated mud; Sandy channel > River
Glycerol	27.613	5.18E-10	2.10E-08	River > Bioturbated mud; Sandy channel > Bioturbated mud; Sandy channel > River
Succinic acid	21.696	2.27E-08	3.50E-07	River > Bioturbated mud; Sandy channel > Bioturbated mud
Quinovose	20.876	3.93E-08	5.54E-07	River > Bioturbated mud; Sandy channel > Bioturbated mud; Sandy channel > River
2′-deoxycytidine	20.602	4.74E-08	6.39E-07	River > Bioturbated mud; River > Sandy channel
2-Amino-2-methyl-1,3-propanediol	17.318	4.66E-07	4.72E-06	River > Bioturbated mud; Sandy channel > Bioturbated mud
Myristic acid	16.621	7.70E-07	7.34E-06	River > Bioturbated mud; River > Sandy channel
D-Melezitose	15.085	2.37E-06	2.14E-05	Sandy channel > Bioturbated mud; Sandy channel > River
Palmitic acid	14.872	2.78E-06	2.20E-05	River > Bioturbated mud; River > Sandy channel
Maltotriose	14.389	3.99E-06	3.01E-05	Sandy channel > Bioturbated mud; Sandy channel > River
Inosine 5′-monophosphate	14.225	4.52E-06	3.25E-05	Bioturbated mud > River; Bioturbated mud > Sandy channel
2-Piperidone	12.784	1.36E-05	7.87E-05	River > Bioturbated mud; River > Sandy channel
Acetylisatin	12.244	2.06E-05	0.00011	Bioturbated mud > Sandy channel; River > Sandy channel
3-Hydroxypropanoic acid	11.937	2.62E-05	0.000131	Bioturbated mud > River; Bioturbated mud > Sandy channel
Caprylic acid	11.391	4.04E-05	0.000192	River > Bioturbated mud; Sandy channel > Bioturbated mud
Capric acid	11.338	4.21E-05	0.000195	River > Bioturbated mud; River > Sandy channel
1,3-Dihydroxyacetone	11.204	4.68E-05	0.000208	Bioturbated mud > River; Bioturbated mud > Sandy channel
Methyl palmitoleate	10.292	9.73E-05	0.000389	Sandy channel > Bioturbated mud; Sandy channel > River
2-Hydroxybutyric acid	10.088	0.000115	0.000438	River > Bioturbated mud; River > Sandy channel
Beta-alanine	10.073	0.000116	0.000438	River > Bioturbated mud; River > Sandy channel
Tartronic acid	9.1039	0.000257	0.000915	Bioturbated mud > River; Bioturbated mud > Sandy channel
1-Methylhydantoin	8.9855	0.000284	0.000988	River > Bioturbated mud; River > Sandy channel
Maltose	8.7579	0.000343	0.001132	Sandy channel > Bioturbated mud; Sandy channel > River
L-Alanine	8.5157	0.000419	0.001294	River > Bioturbated mud; River > Sandy channel
Cellobiose	8.1903	0.000551	0.001622	Sandy channel > Bioturbated mud; Sandy channel > River
Urea	7.9744	0.000661	0.001878	River > Bioturbated mud; Sandy channel > Bioturbated mud
Gluconic acid lactone	7.6863	0.000844	0.002336	River > Bioturbated mud; Sandy channel > Bioturbated mud
Gentisic acid	7.4133	0.001064	0.00285	River > Bioturbated mud; Sandy channel > Bioturbated mud
Muconic acid	7.2306	0.001245	0.003223	Sandy channel > Bioturbated mud; Sandy channel > River
Loganin	7.2223	0.001254	0.003223	River > Bioturbated mud
D-Trehalose	7.1911	0.001288	0.003259	River > Bioturbated mud
L-Pyroglutamic acid	7.0143	0.001499	0.003624	River > Bioturbated mud; River > Sandy channel
Geranial	6.8196	0.001773	0.004224	Sandy channel > Bioturbated mud; Sandy channel > River
Lauric acid	6.7458	0.00189	0.004439	Sandy channel > Bioturbated mud; Sandy channel > River
Scyllo-inositol	6.529	0.002281	0.005241	Sandy channel > Bioturbated mud; Sandy channel > River
Methylmalonic acid	6.1718	0.003115	0.006685	River > Bioturbated mud; River > Sandy channel
Glucoheptonic acid	5.9483	0.00379	0.008027	Sandy channel > Bioturbated mud; Sandy channel > River
Synephrine	5.898	0.003962	0.008336	River > Bioturbated mud; Sandy channel > Bioturbated mud
DL-Glyceraldehyde	5.6602	0.004888	0.009897	River > Bioturbated mud; River > Sandy channel
D-Mannitol	5.6431	0.004962	0.009947	Bioturbated mud > Sandy channel
Tetratriacontane	5.619	0.005069	0.010076	River > Bioturbated mud; River > Sandy channel
Lactic acid	5.5363	0.005455	0.010619	River > Bioturbated mud; River > Sandy channel
Benzoic acid	5.4813	0.005728	0.011046	River > Bioturbated mud
Threose	5.474	0.005765	0.011047	Bioturbated mud > Sandy channel; River > Sandy channel
Geraniol	5.3167	0.006631	0.012419	River > Bioturbated mud; River > Sandy channel
3-Methyl-2-oxobutanoic acid	5.2874	0.006806	0.012468	Sandy channel > Bioturbated mud; Sandy channel > River
3-Phenyllactic acid	4.8964	0.009658	0.016824	Bioturbated mud > Sandy channel
Glycolic acid	4.3905	0.015256	0.02522	River > Bioturbated mud
Methyl hexadecanoate	4.2565	0.017234	0.028344	Sandy channel > Bioturbated mud; Sandy channel > River
Heptadecanoic acid	4.0821	0.020206	0.032899	Sandy channel > River
D-Lyxose	4.0606	0.020607	0.033054	Sandy channel > Bioturbated mud; Sandy channel > River
3,5-Dihydroxyphenylglycine	3.8351	0.025339	0.039661	River > Sandy channel
D-Talose	3.7889	0.026441	0.040601	River > Bioturbated mud; River > Sandy channel
D-Allose	3.7701	0.026901	0.041113	River > Bioturbated mud; River > Sandy channel
2,3-Dihydroxybenzoic acid	3.6152	0.03103	0.046331	River > Bioturbated mud

Seagrass samples were excluded from the analysis as they were under-represented in the sampling of the sites.

**Table 3:** Summary of unique microbial features identified from the different marine sediments sampled from Moreton Bay.

	Class	Order	Family	Genus
**Core depth**				
10 cm	0	2	5	4
20 cm	0	1	1	1
30 cm	1	2	2	6
40 cm	0	1	1	1
**Habitat**				
River	0	1	2	2
Sandy channel	0	1	3	2
Bioturbated mud	1	2	2	5
Seagrass	1	1	2	0

**Table 4:** Summary of taxonomic classification based on core depth bacterial 16S sequencing data from Moreton Bay marine sediment using one-way ANOVA analysis.

Family	*p*-Value	FDR	Fisher’s LSD
*Methylococcaceae*	0.0003	0.0411	20 > 30; 20 > 40; 10 > 30; 10 > 40
*Desulfobulbaceae*	0.0005	0.0411	20 > 30; 20 > 40; 10 > 30; 10 > 40
*Thermoactinomycetaceae*	0.0018	0.0804	30 > 20; 40 > 20; 30 > 10; 40 > 10
*Cystobacteraceae*	0.0021	0.0804	20 > 30; 10 > 30; 10 > 40
*Rhizobiaceae*	0.0029	0.0904	10 > 20; 10 > 30; 10 > 40
*Iamiaceae*	0.0060	0.1520	10 > 20; 10 > 30; 10 > 40
*Kofleriaceae*	0.0074	0.1520	20 > 30; 10 > 30; 10 > 40
*Thermoanaerobacteraceae*	0.0096	0.152	30 > 20; 30 > 10
*Myxococcaceae*	0.0097	0.1520	20 > 30; 20 > 40
*Ectothiorhodospiraceae*	0.0098	0.1520	20 > 30; 10 > 30; 10 > 40
*Caldilineaceae*	0.0149	0.2039	30 > 10; 40 > 10
*Hyphomicrobiaceae*	0.0169	0.2039	10 > 20; 10 > 30; 10 > 40
*Thioalkalispiraceae*	0.0171	0.2039	20 > 30; 20 > 40; 10 > 30; 10 > 40
*Oceanospirillaceae*	0.0204	0.2146	10 > 30; 10 > 40
*Thermotogaceae*	0.0213	0.2146	30 > 20; 30 > 10
*Thermoanaerobacterales Family III. Incertae Sedis*	0.0222	0.2146	40 > 20; 30 > 10; 40 > 10
*Conexibacteraceae*	0.0270	0.2465	10 > 30; 10 > 40
*Chromatiaceae*	0.0333	0.2722	10 > 30; 10 > 40
*Flavobacteriaceae*	0.0334	0.2722	10 > 30; 10 > 40
*Halanaerobiaceae*	0.0381	0.2953	30 > 10; 40 > 10
*Desulfuromonadaceae*	0.0420	0.3101	10 > 30; 10 > 40
*Granulosicoccaceae*	0.0448	0.3153	10 > 30; 10 > 40

**Table 5:** Summary of predicted phenotypic classification based on core depth bacterial 16S sequencing data from Moreton Bay marine sediment using one-way ANOVA analysis.

Type	*p*-Value	FDR	Fisher’s LSD
**Metabolic function**			
Dehalogenation	0.00003	0.0010	20 > 30; 20 > 40; 10 > 30; 10 > 40
Sulfate reducer	0.0001	0.0013	20 > 30; 20 > 40; 10 > 30; 10 > 40
Ammonia oxidizer	0.0006	0.0052	20 > 30; 20 > 40; 10 > 30; 10 > 40
Xylan degrader	0.0012	0.0072	20 > 30; 20 > 40; 10 > 30; 10 > 40
Chitin degradation	0.0020	0.0101	10 > 20; 10 > 30; 10 > 40
Nitrite reducer	0.0053	0.0222	20 > 30; 10 > 30; 10 > 40
Sulfur metabolizing	0.0063	0.0224	20 > 30; 20 > 40; 10 > 30; 10 > 40
Sulfide oxidizer	0.0330	0.0965	10 > 30; 10 > 40
Degrades aromatic hydrocarbons	0.0375	0.0965	20 > 30; 20 > 40; 10 > 30
Syntrophic	0.0415	0.0965	10 > 30; 10 > 40
Atrazine metabolism	0.0425	0.0965	10 > 30
**Oxygen requirements**			
Aerobic	0.0031	0.0140	20 > 30; 10 > 30; 10 > 40
Anaerobic	0.0047	0.0140	30 > 20; 30 > 10, 40 > 10
**Energy Source**			
Methanotroph	0.0002	0.0025	20 > 30; 20 > 40; 10 > 30; 10 > 40
Chemoautotroph	0.0007	0.0055	30 > 20; 30 > 40; 30 > 10; 40 > 10
Phototroph	0.0022	0.0105	10 > 20; 10 > 30; 10 > 40
Methylotroph	0.0026	0.0105	20 > 30; 10 > 30; 10 > 40
Lithoautotroph	0.0127	0.0406	10 > 30; 10 > 40
Organotroph	0.0264	0.0703	30 > 10; 40 > 10

**Table 6:** Summary of taxonomic classification based on habitat bacterial 16S sequencing data from Moreton Bay marine sediment using one-way ANOVA analysis.

Organism	*p* Value	FDR	Fisher’s LSD
*Desulfovibrionaceae*	0.0033	0.1546	Bioturbated mud > Sandy Channel
*Bdellovibrionaceae*	0.0055	0.1546	River > Bioturbated mud; River > Sandy Channel
*Hydrogenophilaceae*	0.0060	0.1546	River > Bioturbated mud; River > Sandy Channel
*Oxalobacteraceae*	0.0067	0.1546	River > Bioturbated mud; River > Sandy Channel
*Acetobacteraceae*	0.0067	0.1546	River > Bioturbated mud; River > Sandy Channel
*Phyllobacteriaceae*	0.0074	0.1546	Bioturbated mud > River; Bioturbated mud > Sandy Channel
*Halothiobacillaceae*	0.0092	0.1546	River > Bioturbated mud; River > Sandy Channel
*Thermaceae*	0.0093	0.1546	Bioturbated mud > River; Bioturbated mud > Sandy Channel
*Nocardioidaceae*	0.0102	0.1546	River > Bioturbated mud; River > Sandy Channel
*Ruminococcaceae*	0.0110	0.1546	Sandy Channel > Bioturbated mud
*Geobacteraceae*	0.0110	0.1546	Bioturbated mud > River; Bioturbated mud > Sandy Channel
*Neisseriaceae*	0.0131	0.1625	River > Bioturbated mud; River > Sandy Channel
*Sinobacteraceae*	0.0168	0.1625	River > Bioturbated mud; River > Sandy Channel
*Natranaerobiaceae*	0.0174	0.1625	Bioturbated mud > River; Bioturbated mud > Sandy Channel
*Syntrophomonadaceae*	0.0179	0.1625	Bioturbated mud > River
*Streptomycetaceae*	0.0198	0.1625	River > Bioturbated mud
*Xanthobacteraceae*	0.0203	0.1625	River > Bioturbated mud; River > Sandy Channel
*Solirubrobacteraceae*	0.0207	0.1625	River > Bioturbated mud; Sandy Channel > Bioturbated mud
*Halomonadaceae*	0.0212	0.1625	Bioturbated mud > Sandy Channel
*Cyclobacteriaceae*	0.0216	0.1625	River > Bioturbated mud; River > Sandy Channel
*Thermithiobacillaceae*	0.0225	0.1625	Bioturbated mud > River; Bioturbated mud > Sandy Channel
*Acidaminococcaceae*	0.0242	0.1625	River > Bioturbated mud; Sandy Channel > Bioturbated mud
*Rhodospirillaceae*	0.0249	0.1625	Bioturbated mud > Sandy Channel
*Paenibacillaceae*	0.0261	0.1625	River > Bioturbated mud; Sandy Channel > Bioturbated mud
*Opitutaceae*	0.0264	0.1625	Bioturbated mud > Sandy Channel; River > Sandy Channel
*Sphingomonadaceae*	0.0283	0.1625	River > Bioturbated mud
*Lachnospiraceae*	0.0295	0.1625	Sandy Channel > Bioturbated mud; Sandy Channel > River
*Helicobacteraceae*	0.0307	0.1625	River > Bioturbated mud; River > Sandy Channel
*Ktedonobacteraceae*	0.0308	0.1625	Sandy Channel > Bioturbated mud; Sandy Channel > River
*Phaselicystidaceae*	0.0315	0.1625	Bioturbated mud > Sandy Channel
*Polyangiaceae*	0.0332	0.1662	Bioturbated mud > River; Bioturbated mud > Sandy Channel
*Rhodocyclaceae*	0.0365	0.1765	River > Sandy Channel
*Oleiphilaceae*	0.0415	0.1950	Bioturbated mud > River; Bioturbated mud > Sandy Channel
*Thermomicrobiaceae*	0.0436	0.1989	River > Bioturbated mud
*Phycisphaeraceae*	0.0455	0.2013	Bioturbated mud > River; Bioturbated mud > Sandy Channel
*Bacillaceae*	0.0472	0.2030	River > Bioturbated mud; Sandy Channel > Bioturbated mud
*Litoricolaceae*	0.0499	0.2090	River > Bioturbated mud

**Table 7:** Summary of predicted phenotypic classification based on habitat bacterial 16S sequencings data from Moreton Bay marine sediment using one-way ANOVA analysis.

Type	*p*-Value	FDR	Fisher’s LSD
**Metabolic function**			
Sulfur oxidizer	0.0055	0.1078	River > Bioturbated mud; River > Sandy Channel
Stores polyhydroxybutyrate	0.0091	0.1078	River > Bioturbated mud; River > Sandy Channel
Cellulose degrader	0.0123	0.1078	Sandy Channel > Bioturbated mud
Lignin degrader	0.0330	0.1851	Bioturbated mud > River; Bioturbated mud > Sandy Channel
Nitrogen fixation	0.0389	0.1851	Sandy Channel > Bioturbated mud
Denitrifying	0.0497	0.1851	River > Bioturbated mud; River > Sandy Channel
**Oxygen requirements**			
Microaerophilic	0.0195	0.1169	Bioturbated mud > Sandy Channel
**Energy Source**			
Photoheterotroph	0.0312	0.4093	River > Bioturbated mud; River > Sandy Channel

## Supplementary Materials

The following are available online at https://www.mdpi.com/2076-2607/7/10/419/s1. Figure S1: Principal Component Analysis (PCA) of Moreton Bay marine sediment site metabolomics data. (A) PCA Score Scatter plot (B) PCA Loading Scatter plot. Figure S2: Partial Least Squares-Discriminant Analysis (PLS-DA) of Moreton Bay marine sediment site metabolomics data. (A) PLS-DA Score Scatter plot, (B) PLS-DA Loading Scatter plot. Figure S3: Orthogonal Partial Least Squares-Discriminant Analysis (OPLS-DA) of Moreton Bay marine sediment site metabolomics data. (A) OPLS-DA Score Scatter plot (B) OPLS-DA Loading Scatter plot. Figure S4: Bacterial classification among core depth sampled sites for the cohort. (A) Class, (B) Order, (C) Genus. Figure S5: Bacterial classification among habitats sampled sites for the cohort. (A) Class, (B) Order, (C) Genus. Table S1: Percentage of sediment grain particles with a diameter smaller than the sieve size fraction indicated for each site. Table S2: Total organic content analysis for each site. Table S3: Metals analyzed by ICP-MS in sampled marine sediments collected from Moreton bay. Table S4: Organic contaminants screened in sampled marine sediments collected from Moreton bay. Table S5: Organic pollutants observed in sampled marine sediments collected from Moreton bay. Table S6: Unidentified significant metabolites from the different marine sediment habitats sampled from Moreton bay. Table S7: Predicted metabolic pathways based on identified metabolites from habitat sediment samples as determined by Fisher’s LSD test.
