# Supplementary material for: Influence of Human Activities on Broad-Scale Estuarine-Marine Habitats Using Omics-Based Approaches Applied to Marine Sediments
Source: Microorganisms. 2019 Oct 4;7(10):419. doi: 10.3390/microorganisms7100419 (PMC6843362; doi:10.3390/microorganisms7100419)
Supplement: Supplementary file 1 [file microorganisms-07-00419-s001.pdf]

Article

Supplementary Section:

Influence of human activities on broadscale estuarine-marine habitats using omics-based approaches applied to marine sediments

Rohan M. Shah[1], Joseph Crosswell[2], Suzanne S. Metcalfe[3], Geoffrey Carlin[2], Paul D. Morrison[4], Avinash V. Karpe[3], Enzo A. Palombo[1], Andy D.L. Steven[2], and David J. Beale[3*]

[1] Department of Chemistry and Biotechnology, Faculty of Science, Engineering and Technology, Swinburne University of Technology, P. O. Box 218, Hawthorn, Victoria 3122, Australia; rshah@swin.edu.au (RMS), epalombo@swin.edu.au (EAP).

[2] Oceans and Atmosphere, Commonwealth Scientific and Industrial Research Organization, P. O. Box 2583, Dutton Park, Queensland 4001, Australia; joey.crosswell@csiro.au (JC); geoffrey.carlin@csiro.au (GC); andy.steven@csiro.au (ADLS).

[3] Land and Water, Commonwealth Scientific and Industrial Research Organization, P. O. Box 2583, Dutton Park, Queensland 4001, Australia; suzanne.metcalfe@csiro.au (SSM); avinash.karpe@csiro.au (AVK); david.beale@csiro.au (DJB).

[4] Australian Centre for Research on Separation Science, School of Applied Sciences, RMIT University, Melbourne, VIC 3001, Australia; paul.morrison@rmit.edu.au (PDM).

* Correspondence: david.beale@csiro.au; Tel.: +61-7-3833-5774

Table 1. Percentage of sediment grain particles with a diameter smaller than the sieve size fraction indicated for each site.

Site	Habitat	Core depth (cm)	Sediment grain particles (%)			
			2.36 < x < 4.76 mm	0.6 < x < 2.36 mm	0.075 < x < 0.6 mm	x < 0.075 mm
			Gravel	Coarse sand	Fine sand	Silt
1	River	10	0.0	51.3	48.7	0.0
		20	0.0	8.4	89.3	2.3
		30	0.1	6.4	91.1	2.4
		40	0.7	8.0	89.8	1.5
2	Sandy channel	10	0.0	21.5	77.0	1.5
		20	1.9	37.3	58.9	2.0
		30	22.0	46.7	27.3	3.9
		40	9.3	37.5	51.2	2.0
3	Sandy channel	10	1.4	2.6	94.9	1.2
		20	0.1	3.6	93.8	2.6
		30	7.4	11.3	75.4	5.8
4	Bioturbed mud	10	0.0	25.1	69.6	5.3
		20	0.0	14.2	77.5	8.3
		30	0.1	1.7	94.7	3.4
		40	0.0	3.6	89.9	6.5
5	Bioturbed mud	10	2.5	17.4	79.3	0.8
		20	1.9	13.8	82.1	2.2
		30	4.8	19.5	75.0	0.8
		40	4.7	9.3	83.9	2.1
6	Bioturbed mud	10	27.0	34.6	33.5	4.9
		20	14.5	17.8	64.2	3.4
		30	13.1	17.4	67.8	1.7
		40	40.3	27.5	31.0	1.3
7	Bioturbed mud	10	8.8	13.4	73.4	4.4
		20	21.1	11.0	67.0	0.9
		30	1.2	4.6	89.4	4.8
8	Seagrass	10	17.9	47.5	34.6	0.0
		20	11.4	37.6	45.7	5.4
9	River	10	1.9	10.4	79.2	8.5
		20	13.5	31.6	51.6	3.3
		30	20.0	26.8	51.6	1.7
		40	0.5	9.1	90.3	0.0

Table 2. Total organic content analysis for each site.

Site	Habitat	Core depth (cm)	Total organic content (mg g^{-1})				Fraction Organic Carbon (f_{OC})
			TC	TOC	IC	TN	
1	River	10	21.48	21.48	0.00	1.57	0.00215
		20	26.24	26.24	0.00	1.95	0.00262
		30	4.53	4.53	0.00	0.25	0.00045
		40	11.81	11.81	0.00	0.60	0.00118
2	Sandy Channel	10	8.35	8.35	0.00	0.60	0.00083
		20	9.22	9.22	0.00	0.54	0.00092
		30	15.52	15.52	0.00	0.88	0.00155
		40	13.92	13.92	0.00	0.86	0.00139
3	Sandy Channel	10	4.65	4.29	0.72	0.30	0.00043
		20	3.84	3.04	0.82	0.17	0.00030
		30	10.47	8.59	2.13	0.66	0.00086
4	Bioturbed mud	10	19.16	11.00	8.56	1.17	0.00110
		20	17.66	9.65	8.33	0.99	0.00097
		30	15.09	6.82	8.41	0.58	0.00068
		40	14.52	5.82	8.78	0.48	0.00058
5	Bioturbed mud	10	22.78	7.27	15.77	1.14	0.00073
		20	23.44	6.57	17.00	1.01	0.00066
		30	23.55	6.17	17.55	0.83	0.00062
		40	22.66	4.92	17.85	0.82	0.00049
6	Bioturbed mud	10	16.75	9.44	7.53	0.95	0.00094
		20	19.41	6.87	12.64	0.99	0.00069
		30	18.86	5.17	13.81	0.78	0.00052
		40	8.65	6.78	1.90	0.78	0.00068
7	Bioturbed mud	10	10.67	8.73	2.15	0.87	0.00087
		20	8.17	6.16	1.96	0.87	0.00062
		30	10.75	9.16	1.59	0.93	0.00092
8	Seagrass	10	62.19	7.55	55.09	1.24	0.00076
		20	15.63	10.70	5.11	1.15	0.00107
9	River	10	8.94	7.57	1.59	1.11	0.00076
		20	12.22	9.71	2.73	1.20	0.00097
		30	9.34	7.48	1.92	0.97	0.00075
		40	2.54	2.07	0.40	0.60	0.00021

TC – Total carbon; TOC – Total organics carbon; IC – Inorganic carbon; TN – Total nitrogen.

Table 3. Metals analyzed by ICP-MS in sampled marine sediments collected from Moreton bay.

			Antimony (Sb)	Cadmium (Cd)	Chromium (Cr)	Copper (Cu)	Lead (Pb)	Nickel (Ni)	Silver (Ag)	Zinc (Zn)
**ISQG-High (trigger value) ***			**25**	**10**	**370**	**270**	**220**	**52**	**3.7**	**410**
**ISQG-Low (trigger value) ***			**2**	**1.5**	**80**	**65**	**50**	**21**	**1**	**200**
**Site**	**Habitat**	**Core depth (cm)**	**Concentration (mg kg^{-1} dry weight)**							
1	River	10	< 0.2 (N.D.)	< 0.1 (N.D.)	1.9 ± < 0.1	1.8 ± < 0.1	0.7 ± 0.2	1.3 ± < 0.1	< 0.1 (N.D.)	4.3 ± < 0.1
		20	< 0.2 (N.D.)	< 0.1 (N.D.)	0.6 ± < 0.1	0.5 ± < 0.1	0.2 ± < 0.1	0.4 ± < 0.1	< 0.1 (N.D.)	1.4 ± 0.4
		30	< 0.2 (N.D.)	< 0.1 (N.D.)	0.7 ± < 0.1	0.7 ± < 0.1	0.3 ± < 0.1	0.5 ± < 0.1	< 0.1 (N.D.)	1.8 ± 0.2
		40	< 0.2 (N.D.)	< 0.1 (N.D.)	1.8 ± < 0.1	1.8 ± < 0.1	0.5 ± < 0.1	1.0 ± < 0.1	< 0.1 (N.D.)	3.6 ± 0.2
2	Sandy Channel	10	< 0.2 (N.D.)	< 0.1 (N.D.)	1.4 ± < 0.1	1.2 ± < 0.1	0.6 ± < 0.1	0.9 ± < 0.1	< 0.1 (N.D.)	3.4 ± 0.2
		20	< 0.2 (N.D.)	< 0.1 (N.D.)	1.2 ± < 0.1	1.1 ± < 0.1	0.5 ± < 0.1	0.9 ± < 0.1	< 0.1 (N.D.)	2.7 ± 0.4
		30	< 0.2 (N.D.)	< 0.1 (N.D.)	1.7 ± < 0.1	1.3 ± < 0.1	0.6 ± < 0.1	1.5 ± 0.1	< 0.1 (N.D.)	3.8 ± 0.5
		40	< 0.2 (N.D.)	< 0.1 (N.D.)	0.8 ± < 0.1	0.8 ± < 0.1	0.2 ± < 0.1	0.7 ± < 0.1	< 0.1 (N.D.)	1.9 ± 0.6
3	Sandy Channel	10	< 0.2 (N.D.)	< 0.1 (N.D.)	0.4 ± < 0.1	0.2 ± < 0.1	0.1 ± < 0.1	0.1 ± < 0.1	< 0.1 (N.D.)	0.4 ± 0.3
		20	< 0.2 (N.D.)	< 0.1 (N.D.)	0.5 ± < 0.1	0.2 ± < 0.1	0.1 ± < 0.1	0.2 ± < 0.1	< 0.1 (N.D.)	0.5 ± 0.4
		30	< 0.2 (N.D.)	< 0.1 (N.D.)	1.7 ± < 0.1	1.4 ± < 0.1	0.4 ± < 0.1	0.7 ± < 0.1	< 0.1 (N.D.)	2.0 ± 0.9

Table 3. Metals analyzed by ICP-MS in sampled marine sediments collected from Moreton bay (*continued*).

			Antimony (Sb)	Cadmium (Cd)	Chromium (Cr)	Copper (Cu)	Lead (Pb)	Nickel (Ni)	Silver (Ag)	Zinc (Zn)
**ISQG-High (trigger value) ***			**25**	**10**	**370**	**270**	**220**	**52**	**3.7**	**410**
**ISQG-Low (trigger value) ***			**2**	**1.5**	**80**	**65**	**50**	**21**	**1**	**200**
**Site**	**Habitat**	**Core depth (cm)**	**Concentration (mg kg^{-1} dry weight)**							
4	Bioturbed mud	10	< 0.2 (N.D.)	< 0.1 (N.D.)	2.2 ± < 0.1	0.9 ± < 0.1	0.7 ± < 0.1	1.1 ± < 0.1	< 0.1 (N.D.)	3.2 ± 0.2
		20	< 0.2 (N.D.)	< 0.1 (N.D.)	2.2 ± < 0.1	1.7 ± < 0.1	0.7 ± < 0.1	1.1 ± < 0.1	< 0.1 (N.D.)	3.2 ± 0.6
		30	< 0.2 (N.D.)	< 0.1 (N.D.)	1.6 ± < 0.1	1.1 ± < 0.1	0.3 ± < 0.1	0.8 ± < 0.1	< 0.1 (N.D.)	2.1 ± 0.7
		40	< 0.2 (N.D.)	< 0.1 (N.D.)	1.6 ± < 0.1	1.1 ± < 0.1	0.4 ± 0.1	0.8 ± < 0.1	< 0.1 (N.D.)	1.9 ± 1.0
5	Bioturbed mud	10	< 0.2 (N.D.)	< 0.1 (N.D.)	1.9 ± < 0.1	0.9 ± < 0.1	0.6 ± 0.2	0.8 ± 0.1	< 0.1 (N.D.)	2.5 ± 0.9
		20	< 0.2 (N.D.)	< 0.1 (N.D.)	1.8 ± < 0.1	0.8 ± < 0.1	0.6 ± < 0.1	0.8 ± < 0.1	< 0.1 (N.D.)	2.3 ± 0.3
		30	< 0.2 (N.D.)	< 0.1 (N.D.)	1.6 ± < 0.1	0.4 ± < 0.1	0.5 ± 0.2	0.7 ± < 0.1	< 0.1 (N.D.)	2.0 ± 0.9
		40	< 0.2 (N.D.)	< 0.1 (N.D.)	1.8 ± < 0.1	0.5 ± < 0.1	0.5 ± < 0.1	0.9 ± < 0.1	< 0.1 (N.D.)	2.2 ± 0.2

*Trigger values taken from the Australian and New Zealand Guidelines for Fresh and Marine Water Quality Management Strategy [26]; N.D. – Not detected

Table 3. Metals analyzed by ICP-MS in sampled marine sediments collected from Moreton bay (*continued*).

			**Antimony (Sb)**	**Cadmium (Cd)**	**Chromium (Cr)**	**Copper (Cu)**	**Lead (Pb)**	**Nickel (Ni)**	**Silver (Ag)**	**Zinc (Zn)**
**ISQG-High (trigger value) ***			**25**	**10**	**370**	**270**	**220**	**52**	**3.7**	**410**
**ISQG-Low (trigger value) ***			**2**	**1.5**	**80**	**65**	**50**	**21**	**1**	**200**
**Site**	**Habitat**	**Core depth (cm)**	**Concentration (mg kg^{-1} dry weight)**							
6	Bioturbed mud	10	< 0.2 (N.D.)	< 0.1 (N.D.)	2.6 ± < 0.1	4.1 ± < 0.1	1.3 ± < 0.1	1.3 ± < 0.1	< 0.1 (N.D.)	5.4 ± 0.6
		20	< 0.2 (N.D.)	< 0.1 (N.D.)	2.1 ± < 0.1	1.9 ± < 0.1	0.5 ± < 0.1	1.2 ± < 0.1	< 0.1 (N.D.)	3.3 ± 0.3
		30	< 0.2 (N.D.)	< 0.1 (N.D.)	1.9 ± < 0.1	0.9 ± < 0.1	0.4 ± < 0.1	1.1 ± < 0.1	< 0.1 (N.D.)	2.8 ± 0.2
		40	< 0.2 (N.D.)	< 0.1 (N.D.)	2.0 ± < 0.1	2.5 ± < 0.1	0.6 ± < 0.1	1.1 ± < 0.1	< 0.1 (N.D.)	2.9 ± 0.7
7	Bioturbed mud	10	< 0.2 (N.D.)	< 0.1 (N.D.)	1.7 ± < 0.1	1.8 ± < 0.1	0.5 ± < 0.1	0.7 ± < 0.1	< 0.1 (N.D.)	1.7 ± 0.7
		20	< 0.2 (N.D.)	< 0.1 (N.D.)	1.3 ± < 0.1	0.9 ± < 0.1	0.4 ± < 0.1	0.5 ± 0.1	< 0.1 (N.D.)	1.2 ± 0.3
		30	< 0.2 (N.D.)	< 0.1 (N.D.)	1.6 ± < 0.1	1.6 ± < 0.1	0.5 ± < 0.1	0.6 ± < 0.1	< 0.1 (N.D.)	1.3 ± 0.1
8	Seagrass	10	< 0.2 (N.D.)	< 0.1 (N.D.)	1.7 ± < 0.1	1.2 ± < 0.1	0.6 ± < 0.1	0.8 ± < 0.1	< 0.1 (N.D.)	2.9 ± 0.8
		20	< 0.2 (N.D.)	< 0.1 (N.D.)	1.8 ± < 0.1	2.0 ± < 0.1	0.6 ± < 0.1	0.7 ± < 0.1	< 0.1 (N.D.)	1.8 ± 0.8

*Trigger values taken from the Australian and New Zealand Guidelines for Fresh and Marine Water Quality Management Strategy [26]; N.D. – Not detected

Table 3. Metals analyzed by ICP-MS in sampled marine sediments collected from Moreton bay (*continued*).

			Antimony (Sb)	Cadmium (Cd)	Chromium (Cr)	Copper (Cu)	Lead (Pb)	Nickel (Ni)	Silver (Ag)	Zinc (Zn)
**ISQG-High (trigger value) ***			**25**	**10**	**370**	**270**	**220**	**52**	**3.7**	**410**
**ISQG-Low (trigger value) ***			**2**	**1.5**	**80**	**65**	**50**	**21**	**1**	**200**
**Site**	**Habitat**	**Core depth (cm)**	**Concentration (mg kg^{-1} dry weight)**							
9	River	10	< 0.2 (N.D.)	< 0.1 (N.D.)	2.1 ± < 0.1	3.3 ± < 0.1	1.2 ± < 0.1	1.3 ± < 0.1	< 0.1 (N.D.)	6.2 ± 0.6
		20	< 0.2 (N.D.)	< 0.1 (N.D.)	2.6 ± < 0.1	2.3 ± < 0.1	4.3 ± < 0.1	1.1 ± < 0.1	< 0.1 (N.D.)	6.1 ± 0.6
		30	< 0.2 (N.D.)	< 0.1 (N.D.)	1.1 ± < 0.1	1.0 ± < 0.1	1.4 ± < 0.1	0.8 ± < 0.1	< 0.1 (N.D.)	4.5 ± 0.9
		40	< 0.2 (N.D.)	< 0.1 (N.D.)	0.7 ± < 0.1	0.3 ± < 0.1	0.4 ± < 0.1	0.6 ± 0.1	1.8 ± 0.3	1.4 ± 1.0

*Trigger values taken from the Australian and New Zealand Guidelines for Fresh and Marine Water Quality Management Strategy [26]; N.D. – Not detected

Table 4. Organic contaminants screened in sampled marine sediments collected from Moreton bay.

Class	Compound	Abbreviation	PubChem CID	Retention Time (min)	Library match (%)
Antibiotic	Clarithromycin	CLA	84029	6.065	94.32
	Penicillin V	PEN	6869	0.98	81.51
	Trimethoprim	TRI	5578	6.633	87.41
Antidepressant	Fluoxetine	FLX	3386	6.096	92.52
Antihistamine	Diphenhydramine	DPHM	3100	5.855	93.82
	Promethazine	PROM	4927	5.956	96.77
Antiseizure	Carbamazepine	CARB	2554	5.732	95.74
Pesticide	N, N-Diethyl-meta-toluamide	DEET	4284	6.547	98.27
	S-methoprene	SMP	1711973	8.614	95.07
Stimulant	Caffeine	CAF	2519	8.414	98.18
β-blocker	DL-Atenolol	ATN	2249	6.003	86.12
	Pindolol	PIN	4828	0.879	86.01
	Propranolol	PRP	4946	1.237	80.74

Table 5. Organic pollutants observed in sampled marine sediments collected from Moreton bay.

Site	Habitat	Core depth (cm)	Pesticides and Personal Care Products (PPCPs), µg g^{-1} TOC dry sediment												
			CLA	PEN	TRI	FLX	DPHM	PROM	CARB	DEET	SMP	CAF	ATN	PIN	PRP
1	River	10	N.D.	N.D.	N.D.	N.D.	N.D.	N.D.	N.D.	33.9	N.D.	N.D.	N.D.	N.D.	N.D.
		20	N.D.	N.D.	N.D.	N.D.	N.D.	N.D.	N.D.	N.D.	N.D.	N.D.	N.D.	N.D.	N.D.
		30	65.4	**81.0**	N.D.	41.1	N.D.	N.D.	67.6	53.2	N.D.	N.D.	**130.3**	N.D.	N.D.
		40	N.D.	N.D.	N.D.	N.D.	N.D.	N.D.	N.D.	N.D.	N.D.	N.D.	47.5	N.D.	N.D.
2	Sandy Channel	10	38.9	30.8	N.D.	N.D.	N.D.	45.1	N.D.	32.4	N.D.	N.D.	75.2	N.D.	N.D.
		20	35.1	N.D.	N.D.	N.D.	N.D.	N.D.	N.D.	31.6	N.D.	N.D.	53.8	N.D.	N.D.
		30	N.D.	N.D.	N.D.	N.D.	N.D.	N.D.	N.D.	N.D.	N.D.	N.D.	N.D.	N.D.	N.D.
		40	N.D.	N.D.	N.D.	N.D.	N.D.	38.2	N.D.	N.D.	N.D.	N.D.	N.D.	N.D.	N.D.
3	Sandy Channel	10	58.4	**80.6**	43.2	37.6	N.D.	**147.8**	N.D.	52.1	N.D.	N.D.	N.D.	N.D.	N.D.
		20	N.D.	69.8	N.D.	54.7	**81.1**	**198.0**	**102.7**	**84.4**	N.D.	N.D.	**201.9**	N.D.	N.D.
		30	32.2	N.D.	N.D.	N.D.	N.D.	**85.8**	N.D.	N.D.	N.D.	N.D.	N.D.	N.D.	N.D.
4	Bioturbed mud	10	N.D.	N.D.	N.D.	N.D.	N.D.	54.2	N.D.	N.D.	N.D.	N.D.	N.D.	N.D.	49.9
		20	36.5	31.0	N.D.	N.D.	N.D.	N.D.	N.D.	N.D.	N.D.	N.D.	60.6	N.D.	N.D.
		30	40.0	N.D.	30.0	34.4	40.5	**101.8**	N.D.	N.D.	N.D.	N.D.	**92.0**	N.D.	N.D.
		40	N.D.	N.D.	34.0	35.7	44.9	**100.4**	N.D.	N.D.	N.D.	N.D.	**92.1**	N.D.	N.D.
5	Bioturbed mud	10	32.7	34.0	N.D.	N.D.	N.D.	N.D.	N.D.	N.D.	N.D.	N.D.	74.0	N.D.	N.D.
		20	50.3	N.D.	N.D.	43.0	N.D.	N.D.	46.8	N.D.	N.D.	N.D.	**95.6**	N.D.	59.4
		30	N.D.	44.4	35.7	N.D.	37.6	**91.4**	N.D.	**89.7**	N.D.	N.D.	68.4	N.D.	67.4
		40	N.D.	44.6	42.3	61.6	N.D.	N.D.	N.D.	N.D.	N.D.	N.D.	**149.7**	N.D.	N.D.
6	Bioturbed mud	10	N.D.	N.D.	N.D.	N.D.	N.D.	72.1	N.D.	N.D.	N.D.	N.D.	50.0	N.D.	N.D.
		20	N.D.	N.D.	N.D.	N.D.	N.D.	N.D.	N.D.	N.D.	N.D.	N.D.	38.6	N.D.	N.D.
		30	71.4	52.5	41.8	N.D.	N.D.	**130.9**	N.D.	44.5	N.D.	N.D.	**101.6**	N.D.	N.D.
		40	53.2	38.9	30.6	N.D.	N.D.	69.9	N.D.	**91.2**	N.D.	31.2	56.8	N.D.	N.D.

N.D. – Not detected.

Table 5. Organic pollutants observed in sampled marine sediments collected from Moreton bay (*continued*).

7	Bioturbed mud	10	N.D.	N.D.	N.D.	N.D.	N.D.	N.D.	N.D.	74.5	N.D.	N.D.	75.3	N.D.	N.D.
		20	N.D.	40.3	N.D.	N.D.	N.D.	N.D.	N.D.	41.7	N.D.	N.D.	53.5	N.D.	N.D.
		30	N.D.	30.0	N.D.	N.D.	N.D.	N.D.	N.D.	62.5	N.D.	N.D.	36.0	N.D.	39.8
8	Seagrass	10	N.D.	N.D.	N.D.	N.D.	N.D.	N.D.	42.0	30.2	N.D.	30.0	71.8	N.D.	42.7
		20	32.8	31.6	N.D.	N.D.	N.D.	N.D.	30.4	N.D.	N.D.	N.D.	32.8	N.D.	42.3
9	River	10	40.7	37.0	N.D.	N.D.	30.0	N.D.	N.D.	30.8	N.D.	N.D.	69.8	N.D.	N.D.
		20	29.9	N.D.	N.D.	N.D.	N.D.	59.9	N.D.	N.D.	N.D.	N.D.	54.5	N.D.	55.7
		30	N.D.	N.D.	N.D.	N.D.	N.D.	N.D.	34.7	N.D.	N.D.	N.D.	51.3	N.D.	31.9
		40	N.D.	**147.3**	N.D.	N.D.	31.5	**378.2**	**143.5**	N.D.	46.2	N.D.	N.D.	N.D.	N.D.

N.D. – Not detected.

Table 6. Unidentified significant metabolites from sampled marine habitat sediments from Moreton bay.

Metabolite	F value	P value	FDR	Fisher's LSD
Compound 31	35.906	4.27E-12	1.31E-09	Sandy channel > Bioturbed mud; Sandy channel > River
Compound 32	29.921	1.29E-10	8.37E-09	Sandy channel > Bioturbed mud; Sandy channel > River
Compound 129	29.577	1.58E-10	8.55E-09	Bioturbed mud > River; Bioturbed mud > Sandy channel
Compound 38	28.595	2.85E-10	1.32E-08	Sandy channel > Bioturbed mud; Sandy channel > River
Compound 274	27.235	6.53E-10	2.12E-08	River > Bioturbed mud; Sandy channel > Bioturbed mud; Sandy channel > River
Compound 296	27.235	6.53E-10	2.12E-08	River > Bioturbed mud; Sandy channel > Bioturbed mud; Sandy channel > River
Compound 99	26.356	1.13E-09	3.32E-08	River > Bioturbed mud; River > Sandy channel
Compound 44	25.644	1.76E-09	4.75E-08	River > Bioturbed mud; Sandy channel > Bioturbed mud; Sandy channel > River
Compound 144	25.032	2.59E-09	6.45E-08	Bioturbed mud > River; Bioturbed mud > Sandy channel
Compound 51	24.181	4.46E-09	1.03E-07	Bioturbed mud > River; Bioturbed mud > Sandy channel
Compound 27	23.225	8.27E-09	1.79E-07	River > Bioturbed mud; Sandy channel > Bioturbed mud; Sandy channel > River
Compound 297	22.717	1.15E-08	2.34E-07	River > Bioturbed mud; Sandy channel > Bioturbed mud
Compound 68	22.237	1.58E-08	2.80E-07	River > Bioturbed mud; Sandy channel > Bioturbed mud
Compound 62	22.237	1.58E-08	2.80E-07	River > Bioturbed mud; Sandy channel > Bioturbed mud
Compound 148	22.18	1.64E-08	2.80E-07	Bioturbed mud > River; Bioturbed mud > Sandy channel
Compound 222	21.794	2.13E-08	3.44E-07	River > Bioturbed mud; Sandy channel > Bioturbed mud
Compound 29	21.104	3.37E-08	4.97E-07	River > Bioturbed mud; Sandy channel > Bioturbed mud; Sandy channel > River
Compound 248	20.291	5.85E-08	7.58E-07	River > Bioturbed mud; Sandy channel > Bioturbed mud; Sandy channel > River
Compound 79	19.576	9.56E-08	1.19E-06	Bioturbed mud > River; Bioturbed mud > Sandy channel
Compound 42	19.139	1.29E-07	1.55E-06	River > Bioturbed mud; Sandy channel > Bioturbed mud; Sandy channel > River
Compound 77	18.983	1.44E-07	1.67E-06	Bioturbed mud > Sandy channel; River > Sandy channel
Compound 109	17.916	3.05E-07	3.41E-06	River > Bioturbed mud; Sandy channel > Bioturbed mud
Compound 281	17.78	3.36E-07	3.62E-06	River > Bioturbed mud; Sandy channel > Bioturbed mud
Compound 102	17.626	3.75E-07	3.92E-06	Bioturbed mud > River; Bioturbed mud > Sandy channel
Compound 130	16.621	7.70E-07	7.34E-06	River > Bioturbed mud; River > Sandy channel
Compound 138	15.684	1.53E-06	1.41E-05	Bioturbed mud > River; Bioturbed mud > Sandy channel
Compound 251	15.037	2.46E-06	2.15E-05	Sandy channel > Bioturbed mud; Sandy channel > River
Compound 263	14.918	2.69E-06	2.20E-05	River > Bioturbed mud; River > Sandy channel

Table 6. Unidentified significant metabolites from sampled marine habitat sediments from Moreton bay (*continued*).

Metabolite	F value	P value	FDR	Fisher's LSD
Compound 286	14.882	2.76E-06	2.20E-05	River > Bioturbed mud; River > Sandy channel
Compound 175	14.873	2.78E-06	2.20E-05	River > Bioturbed mud; River > Sandy channel
Compound 258	14.596	3.42E-06	2.64E-05	Sandy channel > Bioturbed mud; Sandy channel > River
Compound 21	14.301	4.27E-06	3.14E-05	Sandy channel > Bioturbed mud; Sandy channel > River
Compound 170	14.142	4.81E-06	3.39E-05	River > Bioturbed mud; River > Sandy channel
Compound 30	13.315	9.02E-06	6.09E-05	River > Bioturbed mud; Sandy channel > Bioturbed mud
Compound 97	13.144	1.03E-05	6.80E-05	River > Bioturbed mud; Sandy channel > Bioturbed mud
Compound 103	13.091	1.07E-05	6.94E-05	Bioturbed mud > River; Bioturbed mud > Sandy channel; River > Sandy channel
Compound 216	12.955	1.19E-05	7.55E-05	Bioturbed mud > River; Bioturbed mud > Sandy channel
Compound 12	12.92	1.22E-05	7.61E-05	Sandy channel > Bioturbed mud; Sandy channel > River
Compound 260	12.874	1.27E-05	7.74E-05	River > Bioturbed mud; Sandy channel > Bioturbed mud
Compound 196	12.85	1.29E-05	7.74E-05	River > Bioturbed mud; River > Sandy channel
Compound 3	12.781	1.36E-05	7.87E-05	River > Bioturbed mud; River > Sandy channel
Compound 151	12.716	1.43E-05	8.13E-05	Bioturbed mud > River; Bioturbed mud > Sandy channel; River > Sandy channel
Compound 163	12.666	1.49E-05	8.30E-05	River > Bioturbed mud; River > Sandy channel
Compound 150	12.543	1.64E-05	8.98E-05	Bioturbed mud > River; Bioturbed mud > Sandy channel
Compound 149	12.445	1.77E-05	9.53E-05	Bioturbed mud > River; Bioturbed mud > Sandy channel
Compound 110	12.188	2.16E-05	0.000113	River > Bioturbed mud; Sandy channel > Bioturbed mud
Compound 142	12.142	2.24E-05	0.000115	Bioturbed mud > River; Bioturbed mud > Sandy channel
Compound 147	12.013	2.47E-05	0.000125	Bioturbed mud > River; Bioturbed mud > Sandy channel
Compound 262	11.918	2.66E-05	0.000131	River > Bioturbed mud; River > Sandy channel
Compound 288	11.745	3.05E-05	0.000148	Sandy channel > Bioturbed mud; Sandy channel > River
Compound 90	11.338	4.21E-05	0.000195	Bioturbed mud > Sandy channel; River > Sandy channel
Compound 264	11.31	4.30E-05	0.000196	River > Bioturbed mud; River > Sandy channel
Compound 26	11.297	4.35E-05	0.000196	Bioturbed mud > Sandy channel; River > Sandy channel
Compound 197	10.988	5.56E-05	0.000244	River > Bioturbed mud; River > Sandy channel
Compound 132	10.874	6.09E-05	0.000263	Bioturbed mud > River; Bioturbed mud > Sandy channel
Compound 213	10.82	6.36E-05	0.000271	River > Bioturbed mud; Sandy channel > Bioturbed mud

Table 6. Unidentified significant metabolites from sampled marine habitat sediments from Moreton bay (*continued*).

Metabolite	F value	P value	FDR	Fisher's LSD
Compound 91	10.614	7.50E-05	0.000316	River > Bioturbed mud; Sandy channel > Bioturbed mud; Sandy channel > River
Compound 114	10.474	8.40E-05	0.000349	Bioturbed mud > River; Bioturbed mud > Sandy channel
Compound 204	10.372	9.12E-05	0.000374	River > Bioturbed mud; River > Sandy channel
Compound 202	10.357	9.23E-05	0.000374	River > Bioturbed mud; River > Sandy channel
Compound 39	10.189	0.000106	0.000418	River > Bioturbed mud; Sandy channel > Bioturbed mud
Compound 89	10.138	0.00011	0.00043	Sandy channel > Bioturbed mud; Sandy channel > River
Compound 161	10.08	0.000116	0.000438	River > Bioturbed mud; Sandy channel > Bioturbed mud
Compound 164	9.9981	0.000123	0.00046	River > Bioturbed mud; River > Sandy channel
Compound 123	9.6182	0.000168	0.00062	River > Bioturbed mud; Sandy channel > Bioturbed mud
Compound 137	9.5756	0.000174	0.000635	River > Bioturbed mud; River > Sandy channel
Compound 206	9.2003	0.000237	0.000855	River > Bioturbed mud; River > Sandy channel
Compound 47	9.0181	0.000276	0.000972	River > Bioturbed mud; Sandy channel > Bioturbed mud
Compound 40	8.9567	0.00029	0.001001	Bioturbed mud > River; Bioturbed mud > Sandy channel
Compound 80	8.8773	0.00031	0.001047	River > Bioturbed mud; Sandy channel > Bioturbed mud
Compound 23	8.877	0.00031	0.001047	Sandy channel > Bioturbed mud; Sandy channel > River
Compound 100	8.8416	0.000319	0.001067	River > Bioturbed mud; Sandy channel > Bioturbed mud
Compound 256	8.717	0.000354	0.001158	Sandy channel > Bioturbed mud; Sandy channel > River
Compound 227	8.7065	0.000357	0.001158	River > Bioturbed mud; Sandy channel > Bioturbed mud
Compound 11	8.6814	0.000365	0.001171	River > Bioturbed mud; River > Sandy channel
Compound 141	8.6584	0.000372	0.001182	Bioturbed mud > Sandy channel; River > Sandy channel
Compound 128	8.6021	0.00039	0.001227	Bioturbed mud > River; Bioturbed mud > Sandy channel
Compound 81	8.5368	0.000412	0.001283	River > Bioturbed mud; River > Sandy channel
Compound 72	8.4833	0.000431	0.001317	River > Bioturbed mud; Sandy channel > Bioturbed mud
Compound 257	8.3711	0.000473	0.001433	Bioturbed mud > River; Sandy channel > River
Compound 10	8.3554	0.000479	0.001438	River > Bioturbed mud; River > Sandy channel
Compound 232	8.2108	0.000541	0.001609	Sandy channel > Bioturbed mud; Sandy channel > River
Compound 233	8.1704	0.00056	0.001635	Bioturbed mud > River; Sandy channel > River
Compound 254	8.0185	0.000637	0.001842	River > Bioturbed mud; Sandy channel > Bioturbed mud

Table 6. Unidentified significant metabolites from sampled marine habitat sediments from Moreton bay (*continued*).

Metabolite	F value	P value	FDR	Fisher's LSD
Compound 166	7.9946	0.00065	0.001863	River > Bioturbed mud; River > Sandy channel
Compound 279	7.9348	0.000683	0.001909	Sandy channel > Bioturbed mud; Sandy channel > River
Compound 280	7.9348	0.000683	0.001909	Sandy channel > Bioturbed mud; Sandy channel > River
Compound 121	7.5975	0.00091	0.002498	Sandy channel > Bioturbed mud; Sandy channel > River
Compound 295	7.5583	0.000941	0.002561	River > Bioturbed mud; Sandy channel > Bioturbed mud
Compound 9	7.4635	0.00102	0.002754	River > Bioturbed mud; River > Sandy channel
Compound 168	7.3904	0.001086	0.002883	River > Bioturbed mud; River > Sandy channel
Compound 82	7.2349	0.00124	0.003223	Sandy channel > Bioturbed mud
Compound 122	7.2248	0.001251	0.003223	Sandy channel > Bioturbed mud; Sandy channel > River
Compound 236	7.1984	0.00128	0.003259	River > Bioturbed mud; Sandy channel > Bioturbed mud
Compound 70	7.167	0.001315	0.003301	Sandy channel > Bioturbed mud
Compound 209	7.1361	0.00135	0.00334	Sandy channel > Bioturbed mud; Sandy channel > River
Compound 211	7.1356	0.00135	0.00334	Sandy channel > Bioturbed mud; Sandy channel > River
Compound 208	7.1205	0.001368	0.003358	Sandy channel > Bioturbed mud; Sandy channel > River
Compound 78	7.095	0.001398	0.003406	Bioturbed mud > River; Bioturbed mud > Sandy channel
Compound 19	6.8789	0.001684	0.004043	River > Bioturbed mud; River > Sandy channel
Compound 92	6.7454	0.001891	0.004439	River > Bioturbed mud; Sandy channel > Bioturbed mud
Compound 265	6.6265	0.002096	0.004885	River > Bioturbed mud; River > Sandy channel
Compound 165	6.6063	0.002133	0.004936	River > Bioturbed mud; River > Sandy channel
Compound 224	6.5208	0.002297	0.005242	Sandy channel > Bioturbed mud; Sandy channel > River
Compound 33	6.4699	0.002402	0.005441	River > Bioturbed mud
Compound 101	6.4576	0.002427	0.005461	Sandy channel > Bioturbed mud; Sandy channel > River
Compound 98	6.4243	0.002499	0.005583	River > Bioturbed mud; Sandy channel > Bioturbed mud
Compound 59	6.41	0.00253	0.005615	Sandy channel > Bioturbed mud; Sandy channel > River
Compound 223	6.3529	0.002659	0.005861	Bioturbed mud > River
Compound 95	6.3336	0.002705	0.005921	River > Bioturbed mud; Sandy channel > Bioturbed mud
Compound 237	6.3206	0.002735	0.005948	River > Bioturbed mud
Compound 243	6.2171	0.002994	0.006468	Bioturbed mud > River

Table 6. Unidentified significant metabolites from sampled marine habitat sediments from Moreton bay (*continued*).

Metabolite	F value	P value	FDR	Fisher's LSD
Compound 96	6.1612	0.003144	0.006702	River > Bioturbed mud; Sandy channel > Bioturbed mud
Compound 56	5.8076	0.004291	0.008908	Bioturbed mud > Sandy channel
Compound 278	5.8026	0.00431	0.008908	River > Bioturbed mud; Sandy channel > Bioturbed mud
Compound 301	5.8007	0.004317	0.008908	River > Bioturbed mud; Sandy channel > Bioturbed mud
Compound 58	5.7936	0.004344	0.008908	Bioturbed mud > Sandy channel; River > Sandy channel
Compound 272	5.6796	0.004804	0.00979	River > Bioturbed mud
Compound 53	5.6405	0.004974	0.009947	Bioturbed mud > Sandy channel
Compound 107	5.5777	0.005258	0.010387	River > Bioturbed mud
Compound 94	5.5561	0.00536	0.010524	River > Bioturbed mud; Sandy channel > Bioturbed mud
Compound 104	5.5324	0.005473	0.010619	Sandy channel > Bioturbed mud
Compound 238	5.4678	0.005796	0.011047	River > Bioturbed mud
Compound 108	5.3842	0.006244	0.01183	Sandy channel > Bioturbed mud
Compound 5	5.3294	0.006556	0.012349	Sandy channel > Bioturbed mud; Sandy channel > River
Compound 86	5.2928	0.006773	0.012468	Sandy channel > Bioturbed mud; Sandy channel > River
Compound 87	5.2928	0.006773	0.012468	Sandy channel > Bioturbed mud; Sandy channel > River
Compound 245	5.2866	0.006811	0.012468	Sandy channel > Bioturbed mud
Compound 93	5.276	0.006876	0.012515	River > Bioturbed mud; Sandy channel > Bioturbed mud
Compound 120	5.2605	0.006972	0.012619	Sandy channel > Bioturbed mud
Compound 45	5.188	0.007438	0.013388	River > Bioturbed mud; River > Sandy channel
Compound 140	5.1279	0.007848	0.014049	River > Bioturbed mud; River > Sandy channel
Compound 244	5.1126	0.007956	0.014164	Sandy channel > Bioturbed mud
Compound 250	5.0157	0.008677	0.015326	Sandy channel > Bioturbed mud; Sandy channel > River
Compound 152	5.0123	0.008704	0.015326	Bioturbed mud > Sandy channel
Compound 156	4.9996	0.008804	0.015418	Sandy channel > Bioturbed mud; Sandy channel > River
Compound 119	4.8452	0.010114	0.017523	Bioturbed mud > Sandy channel; River > Sandy channel
Compound 291	4.6117	0.012485	0.021516	River > Bioturbed mud
Compound 269	4.5736	0.012922	0.022153	River > Bioturbed mud
Compound 285	4.5125	0.013658	0.02329	River > Bioturbed mud

Table 6. Unidentified significant metabolites from sampled marine habitat sediments from Moreton bay (*continued*).

Metabolite	F value	P value	FDR	Fisher's LSD
Compound 282	4.501	0.013801	0.023411	Bioturbed mud > River
Compound 63	4.4284	0.014739	0.024784	River > Sandy channel
Compound 229	4.4266	0.014763	0.024784	Bioturbed mud > River; Sandy channel > River
Compound 116	4.4189	0.014868	0.02483	River > Bioturbed mud; Sandy channel > Bioturbed mud
Compound 112	4.4052	0.015054	0.025012	Bioturbed mud > River; Bioturbed mud > Sandy channel
Compound 126	4.2156	0.017888	0.029271	Bioturbed mud > Sandy channel
Compound 4	4.0693	0.020443	0.032965	River > Bioturbed mud
Compound 74	4.069	0.020451	0.032965	Bioturbed mud > River
Compound 34	4.0462	0.02088	0.033325	River > Bioturbed mud
Compound 117	3.9266	0.023299	0.037004	Bioturbed mud > Sandy channel
Compound 49	3.9126	0.0236	0.037293	River > Bioturbed mud; Sandy channel > Bioturbed mud
Compound 172	3.9075	0.023711	0.037293	Sandy channel > Bioturbed mud; Sandy channel > River
Compound 35	3.8188	0.025722	0.040066	River > Bioturbed mud
Compound 230	3.7975	0.026231	0.040601	Sandy channel > Bioturbed mud; Sandy channel > River
Compound 268	3.7917	0.026371	0.040601	River > Bioturbed mud
Compound 235	3.7515	0.027367	0.041628	River > Bioturbed mud; Sandy channel > Bioturbed mud
Compound 2	3.7451	0.027528	0.041678	Sandy channel > Bioturbed mud; Sandy channel > River
Compound 113	3.7038	0.028594	0.04309	Bioturbed mud > River
Compound 210	3.6158	0.031011	0.046331	River > Bioturbed mud; River > Sandy channel
Compound 190	3.5683	0.032402	0.048158	Bioturbed mud > Sandy channel; River > Sandy channel

The seagrass samples were excluded from the analysis as they were under-represented in the sampling of the sites.

Table 7. Predicted metabolic pathways based on identified metabolites from habitat sediment samples as determined by Fisher's LSD test.

Metabolic Pathway	Total	Expected	Hits	P value	Impact
**Bioturbed mud**					
Glycerolipid metabolism	14	0.13848	1	0.1307	0.13043
Fructose and mannose metabolism	30	0.29674	1	0.2611	< 0.0001
Purine metabolism	73	0.72206	1	0.5290	0.10253
**River**					
Benzoate degradation via CoA ligation	10	0.27695	2	0.0290	< 0.0001
D-Alanine metabolism	3	0.083086	1	0.0809	< 0.0001
Propanoate metabolism	20	0.55391	2	0.1034	0.05405
Biosynthesis of siderophore group nonribosomal peptides	4	0.11078	1	0.1064	< 0.0001
Biosynthesis of unsaturated fatty acids	6	0.16617	1	0.1554	< 0.0001
Glyoxylate and dicarboxylate metabolism	29	0.80317	2	0.1901	0.09464
Starch and sucrose metabolism	31	0.85856	2	0.2106	0.0954
Tyrosine metabolism	10	0.27695	1	0.2458	< 0.0001
Glycerolipid metabolism	14	0.38773	1	0.3269	0.21739
beta-Alanine metabolism	16	0.44313	1	0.3642	0.69231
Alanine, aspartate and glutamate metabolism	18	0.49852	1	0.3995	< 0.0001
Selenoamino acid metabolism	18	0.49852	1	0.3995	< 0.0001
Butanoate metabolism	18	0.49852	1	0.3995	0.02941
Citrate cycle (TCA cycle)	20	0.55391	1	0.4329	0.0372
Phenylalanine metabolism	23	0.63699	1	0.4796	< 0.0001
Pantothenate and CoA biosynthesis	23	0.63699	1	0.4796	0.02417
Pentose phosphate pathway	26	0.72008	1	0.5227	< 0.0001
Pyruvate metabolism	26	0.72008	1	0.5227	0.07336
Galactose metabolism	37	1.0247	1	0.6530	< 0.0001
Fatty acid metabolism	41	1.1355	1	0.6913	< 0.0001
Pyrimidine metabolism	44	1.2186	1	0.7173	0.00607
Aminoacyl-tRNA biosynthesis	66	1.8279	1	0.8529	< 0.0001

Table 7. Predicted metabolic pathways based on identified metabolites from habitat sediment samples as determined by Fisher's LSD test (*continued*).

Metabolic Pathway	Total	Expected	Hits	P value	Impact
**Sandy channel**					
Valine, leucine and isoleucine degradation	23	0.4095	2	0.0608	< 0.0001
Pantothenate and CoA biosynthesis	23	0.4095	2	0.0608	0.08524
Valine, leucine and isoleucine biosynthesis	26	0.46291	2	0.0756	0.10594
Tyrosine metabolism	10	0.17804	1	0.1651	< 0.0001
Benzoate degradation via CoA ligation	10	0.17804	1	0.1651	< 0.0001
Glycerolipid metabolism	14	0.24926	1	0.2237	0.21739
Alanine, aspartate and glutamate metabolism	18	0.32047	1	0.2783	< 0.0001
Butanoate metabolism	18	0.32047	1	0.2783	0.02941
Citrate cycle (TCA cycle)	20	0.35608	1	0.3042	0.0372
Propanoate metabolism	20	0.35608	1	0.3042	0.05405
Phenylalanine metabolism	23	0.4095	1	0.3415	< 0.0001
Pentose phosphate pathway	26	0.46291	1	0.3769	< 0.0001
Glyoxylate and dicarboxylate metabolism	29	0.51632	1	0.4105	< 0.0001
Galactose metabolism	37	0.65875	1	0.4918	< 0.0001
Aminoacyl-tRNA biosynthesis	66	1.1751	1	0.7065	< 0.0001

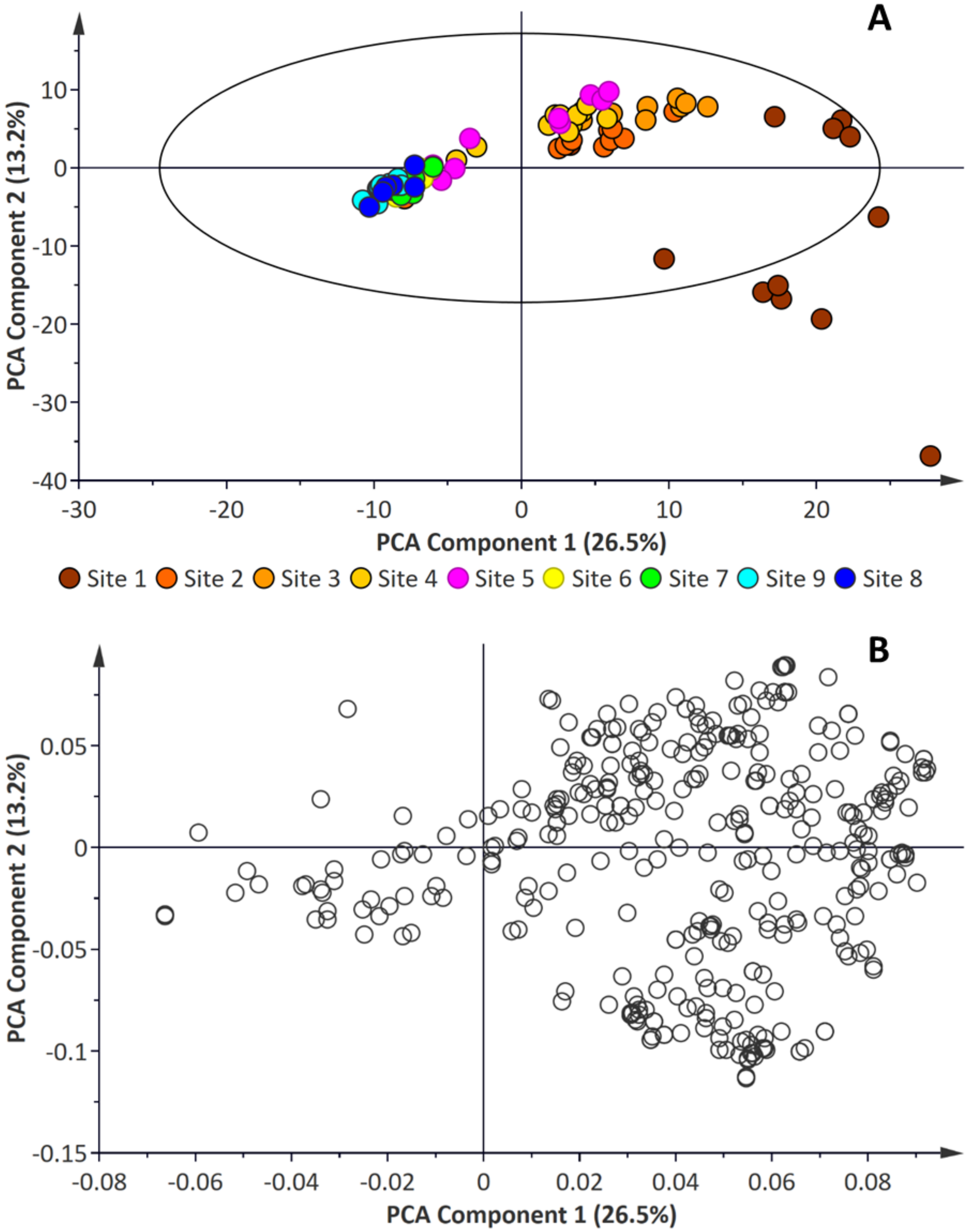

Figure 1. Principal Component Analysis (PCA) of Moreton Bay marine sediment site metabolomics data A. PCA Score Scatter plot, B. PCA Loading Scatter plot. Note: the PCA model linearity (R2X) and predictability (Q2) were observed at 0.814 and 0.464, respectively. This is indicative of a good fit model (> 0.7) and has a poor predictive capability (<0.5).

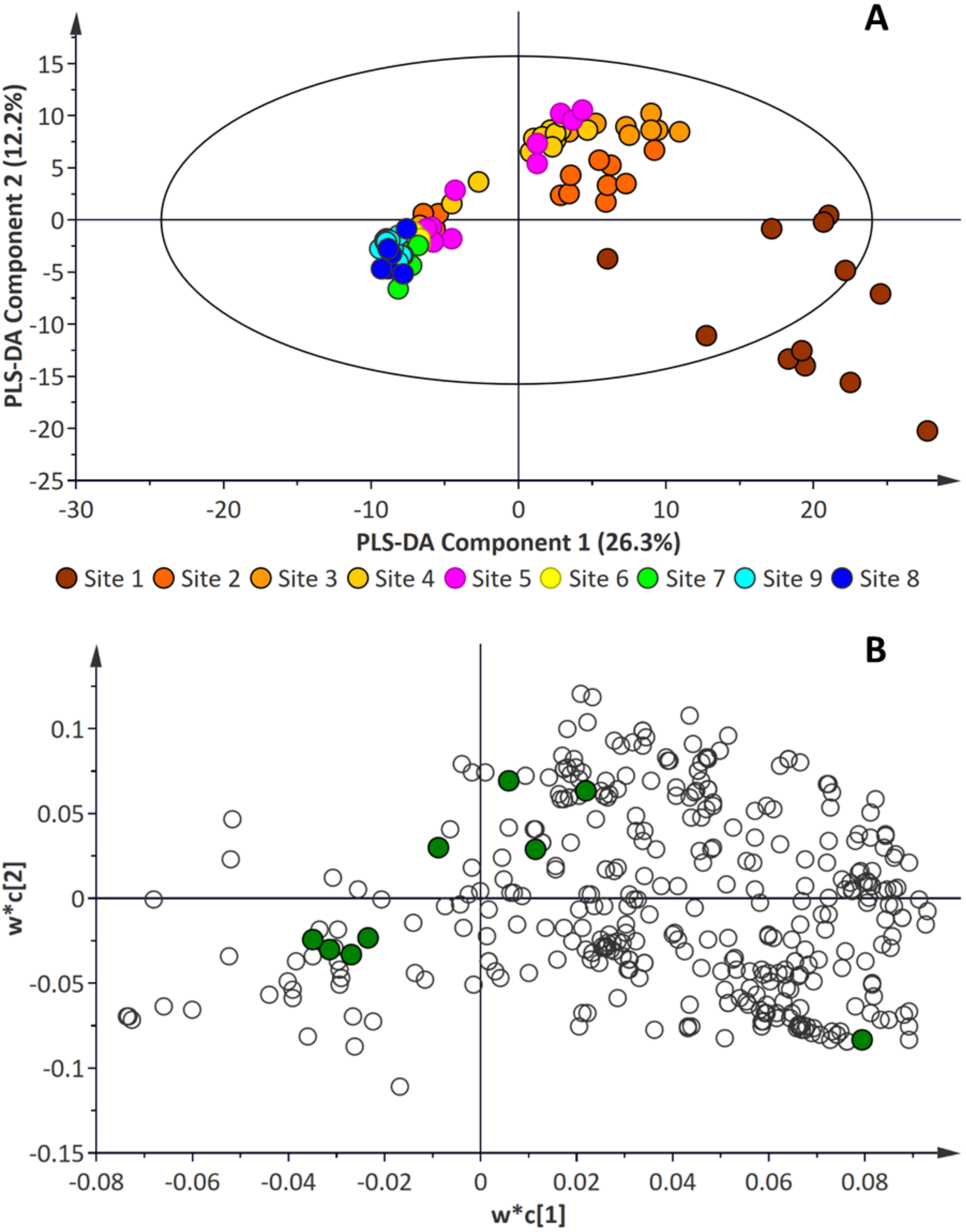

Figure 2. Partial Least Squares – Discriminant Analysis (PLS-DA) of Moreton Bay marine sediment site metabolomics data A. PLS-DA Score Scatter plot, B. PLS-DA Loading Scatter plot. Note: the PLS-DA model linearity (R2X and R2Y) and predictability (Q2) were observed at 0.837, 0.904 and 0.541, respectively. This is indicative of a good fit model (> 0.7) and has a poor predictive capability (~0.5).

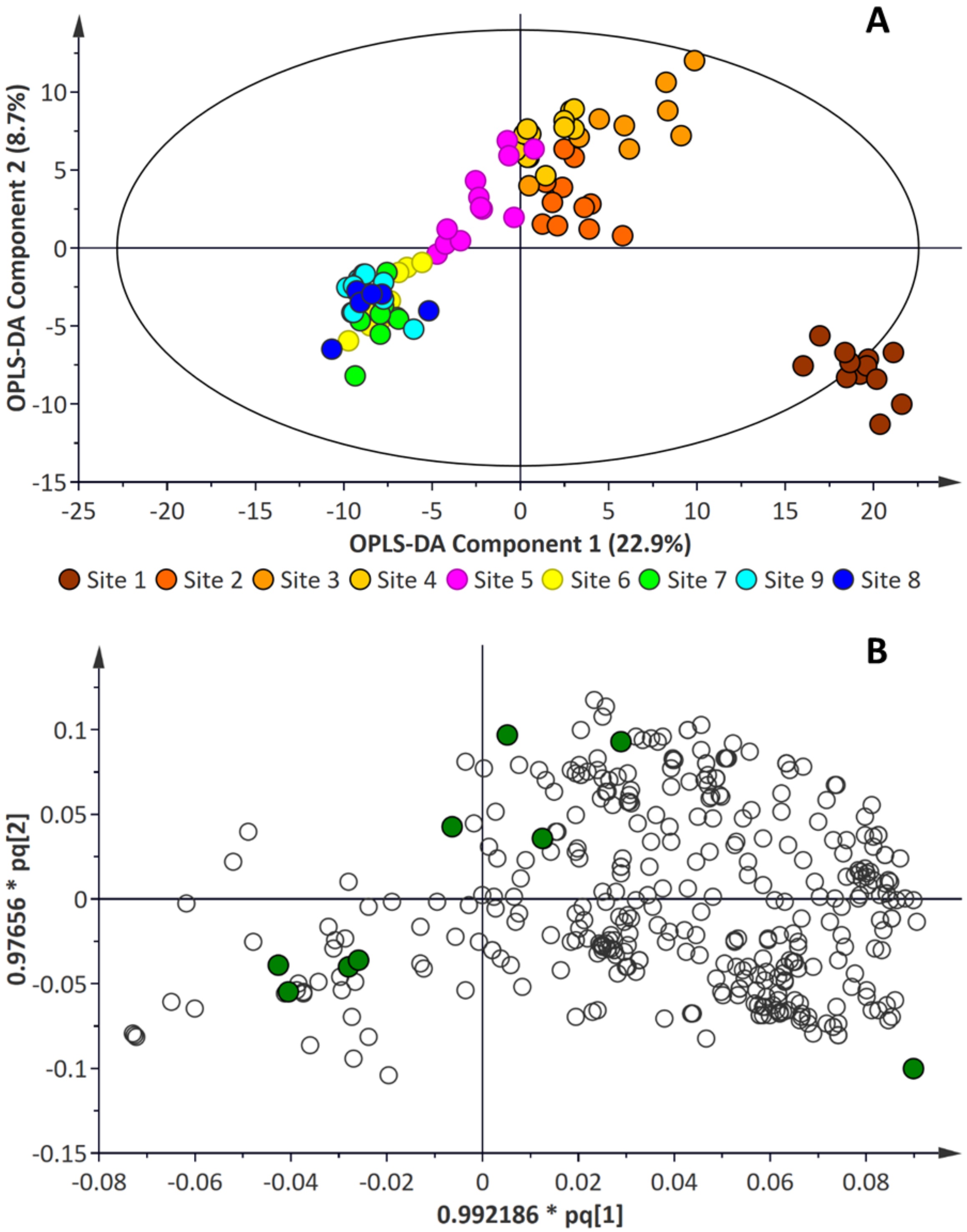

Figure 3. Orthogonal Partial Least Squares – Discriminant Analysis (OPLS-DA) of Moreton Bay marine sediment site metabolomics data A. OPLS-DA Score Scatter plot, B. OPLS-DA Loading Scatter plot. Note: the OPLS-DA model linearity (R2X and R2Y) and predictability (Q2) were observed at 0.726, 0.769 and 0.562, respectively. This is indicative of a good fit model (> 0.7) and has a poor predictive capability (~0.5).

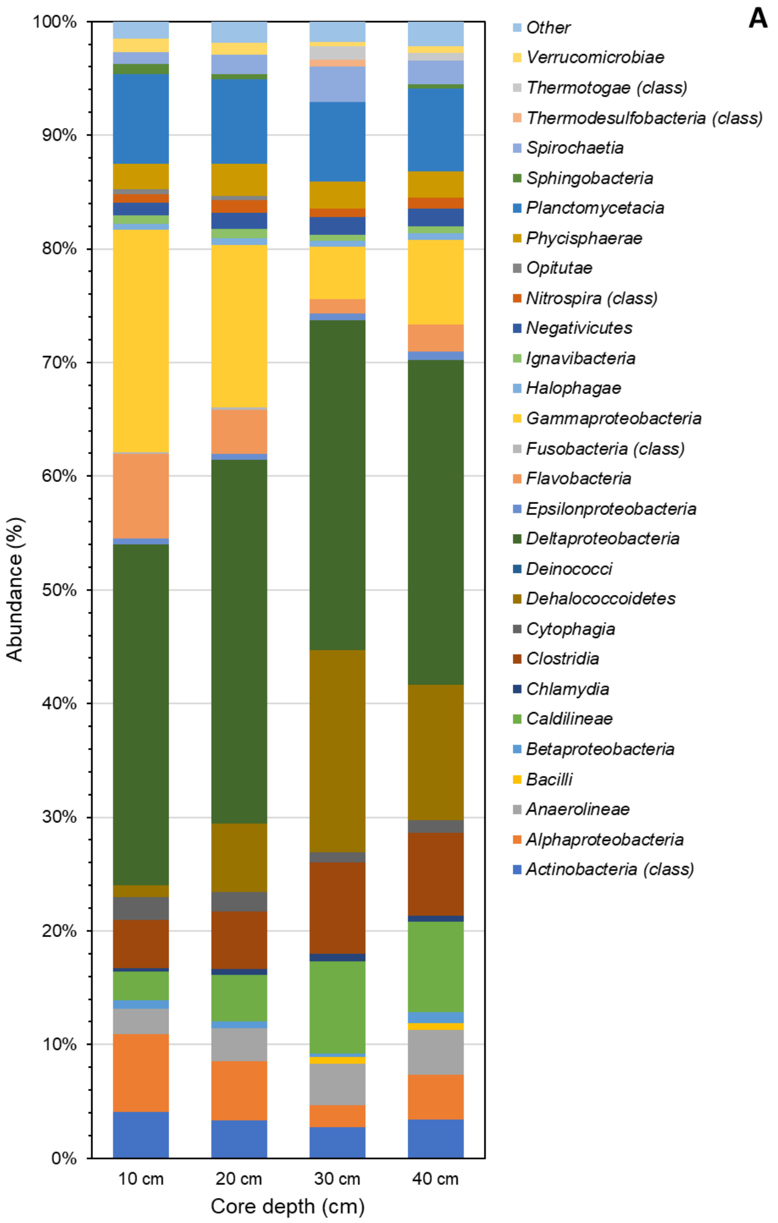

Figure 4. Bacterial classification among core depth sampled sites for cohort A. Class, B. Order, C. Genus.

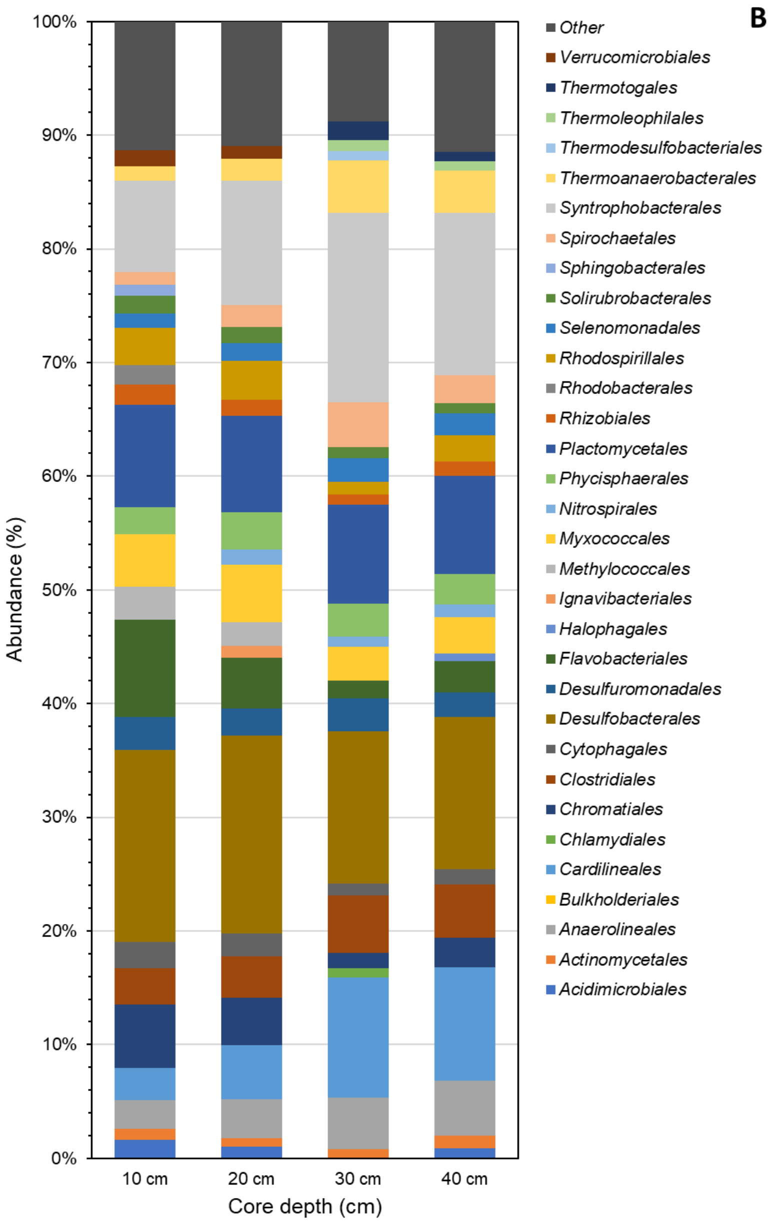

Figure 4. Bacterial classification among core depth sampled sites for cohort A. Class, B. Order, C. Genus (*continued*).

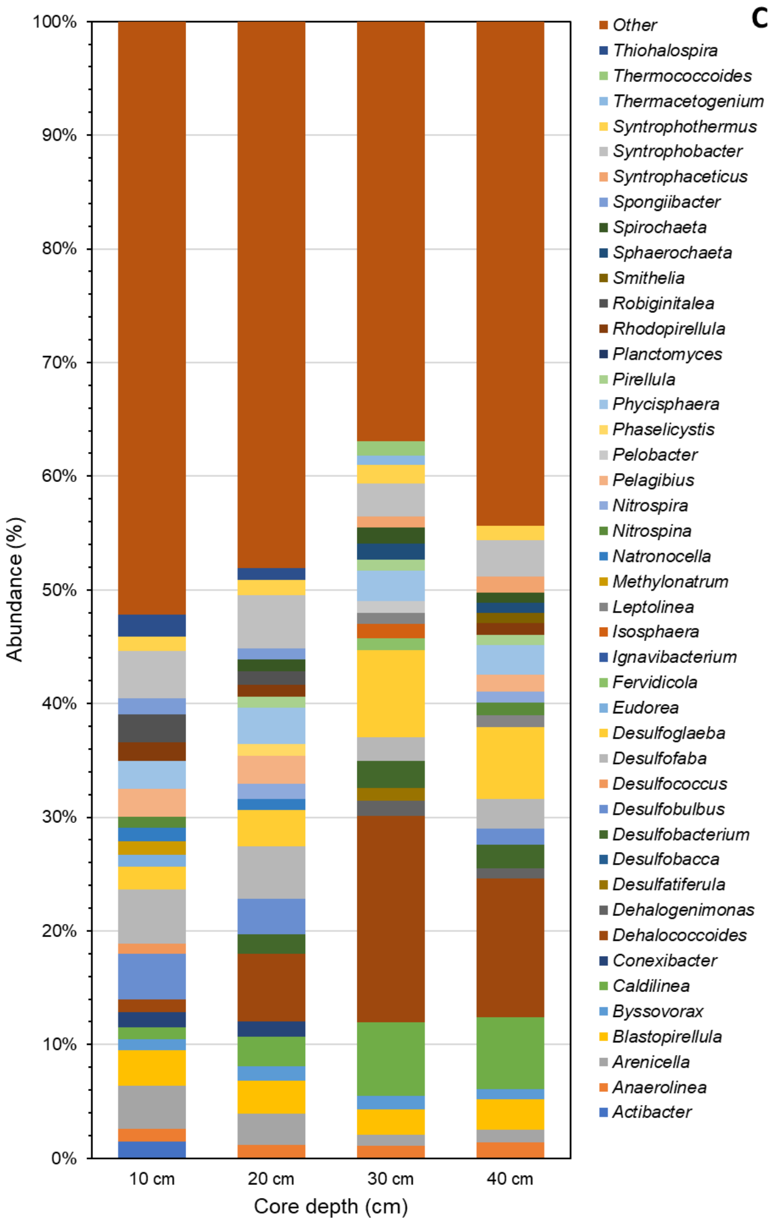

Figure 4. Bacterial classification among core depth sampled sites for cohort A. Class, B. Order, C. Genus (*continued*).

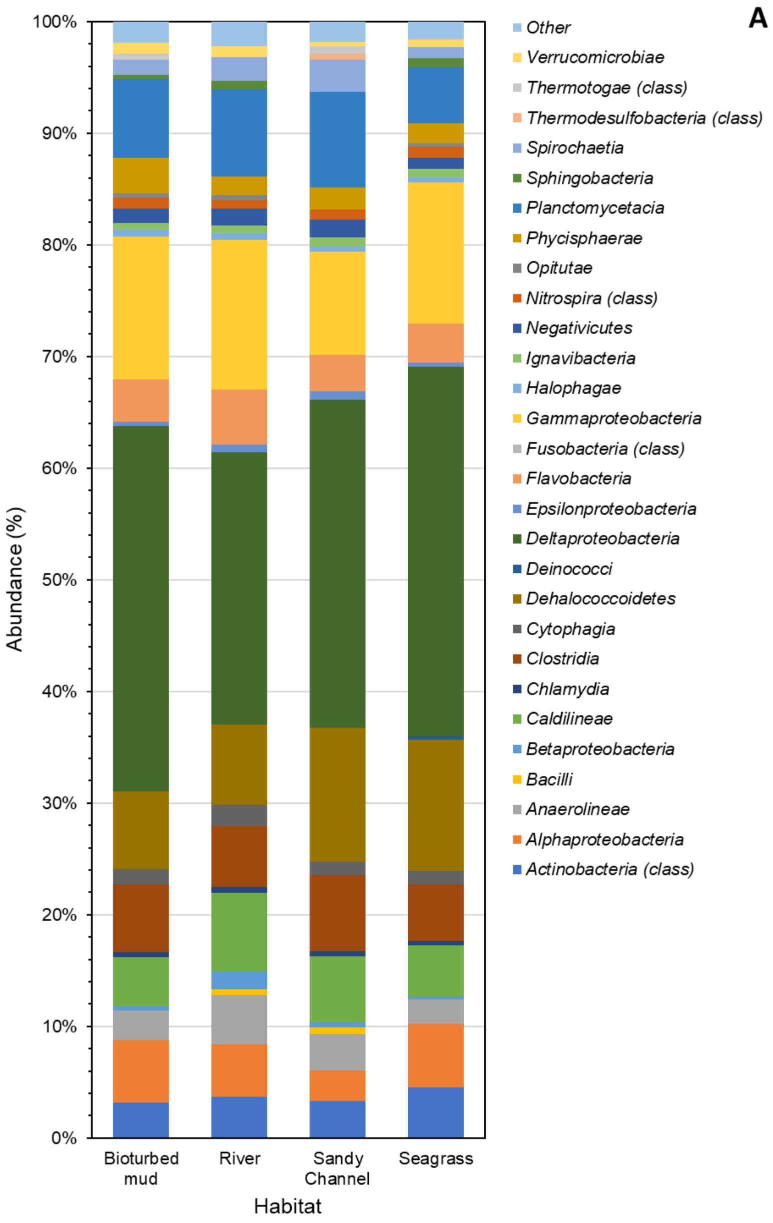

Figure 5. Bacterial classification among habitats sampled sites for cohort A. Class, B. Order, C. Genus (continued).

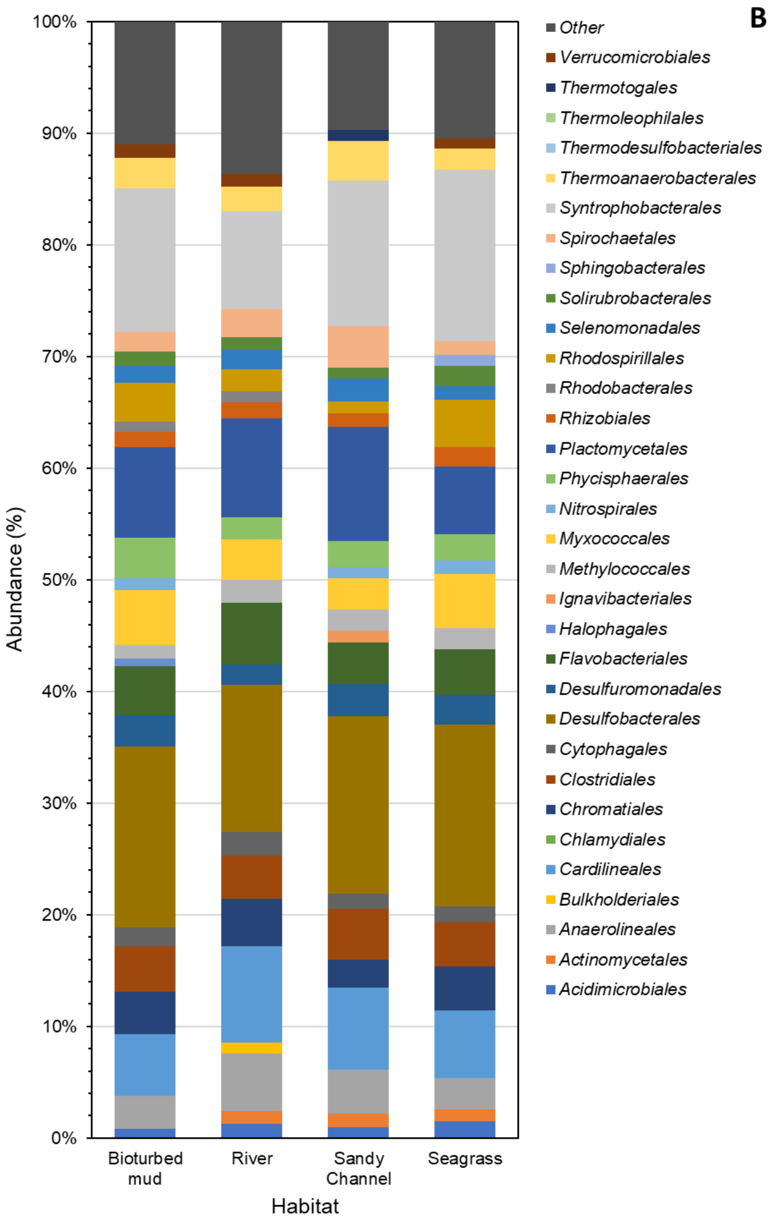

Figure 5. Bacterial classification among habitats sampled sites for cohort A. Class, B. Order, C. Genus (*continued*).

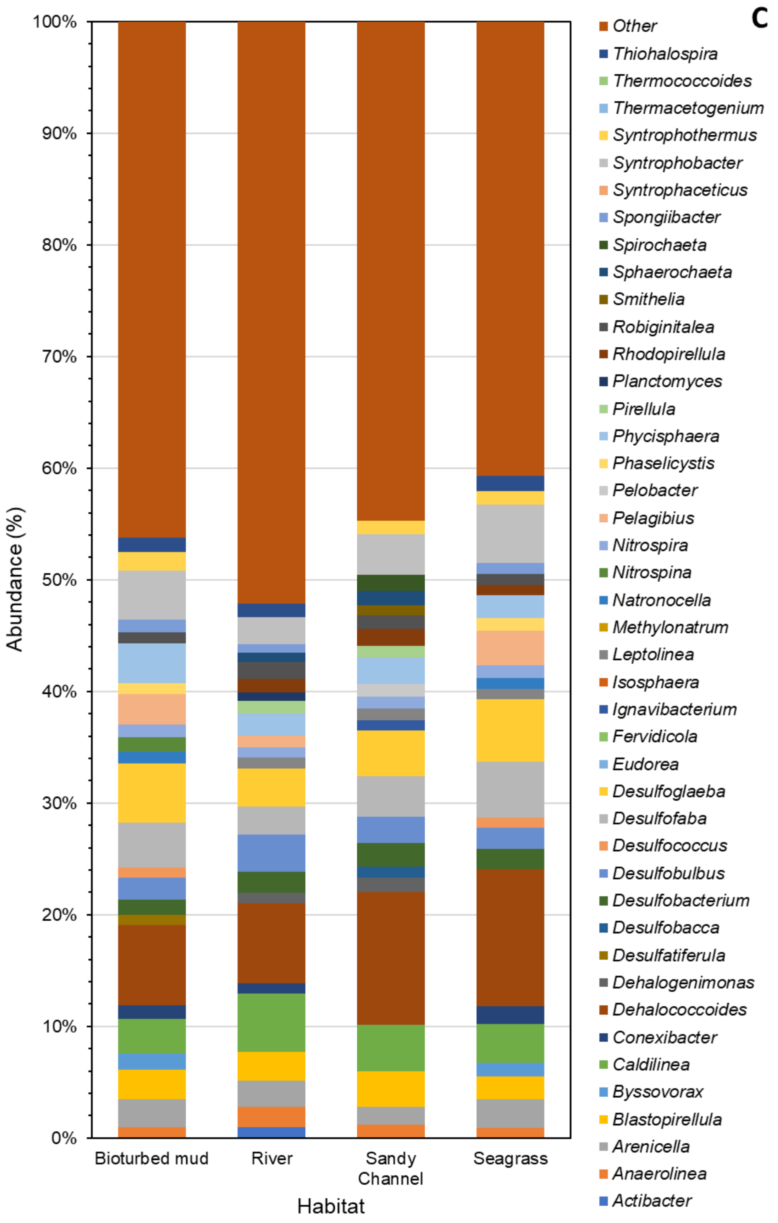

Figure 5. Bacterial classification among habitats sampled sites for cohort A. Class, B. Order, C. Genus (*continued*).
